# Massively parallel characterization of CRISPR activator efficacy in human induced pluripotent stem cells and neurons

**DOI:** 10.1016/j.molcel.2023.02.011

**Published:** 2023-04-06

**Authors:** Qianxin Wu, Junjing Wu, Kaiser Karim, Xi Chen, Tengyao Wang, Sho Iwama, Stefania Carobbio, Peter Keen, Antonio Vidal-Puig, Mark R. Kotter, Andrew Bassett

**Affiliations:** 1 Wellcome Sanger Institute, Hinxton, Cambridge CB10 1SA, UK; 2 Department of Clinical Neurosciences, University of Cambridge, Cambridge CB2 0QQ, UK; 3 Institute of Animal Science and Veterinary Medicine, Hubei Academy of Agricultural Sciences, Wuhan 430064, China; 4 Southern University of Science and Technology, 1088 Xueyuan Ave, Nanshan, Shenzhen, Guangdong 518055, China; 5 Department of Statistics, London School of Economics and Political Science, London WC2B 4RR, UK; 6 Metabolic Research Laboratories, Addenbrooke’s Treatment Center, Institute of Metabolic Science, Addenbrooke’s Hospital, University of Cambridge, Cambridge, UK; 7 Centro de Investigacion Principe Felipe, 46012 Valencia, Spain

**Keywords:** CRISPR activation, CRISPRa, VPR, p300, epigenetic, chromatin, single cell, hiPSC, stem cells, iNeurons

## Abstract

CRISPR activation (CRISPRa) is an important tool to perturb transcription, but its effectiveness varies between target genes. We employ human pluripotent stem cells with thousands of randomly integrated barcoded reporters to assess epigenetic features that influence CRISPRa efficacy. Basal expression levels are influenced by genomic context and dramatically change during differentiation to neurons. Gene activation by dCas9-VPR is successful in most genomic contexts, including developmentally repressed regions, and activation level is anti-correlated with basal gene expression, whereas dCas9-p300 is ineffective in stem cells. Certain chromatin states, such as bivalent chromatin, are particularly sensitive to dCas9-VPR, whereas constitutive heterochromatin is less responsive. We validate these rules at endogenous genes and show that activation of certain genes elicits a change in the stem cell transcriptome, sometimes showing features of differentiated cells. Our data provide rules to predict CRISPRa outcome and highlight its utility to screen for factors driving stem cell differentiation.

## Highlights

• Analysis of CRISPRa activity in hPSCs with thousands of barcoded reporters
• The efficacy of CRISPRa depends on basal expression and chromatin state
• Bivalent genes are more sensitive to CRISPRa and H3K9me3 chromatin is less responsive
• Single-cell CRISPRa screen validates chromatin-dependent effect at endogenous genes

## Graphical abstract

**Figure:**
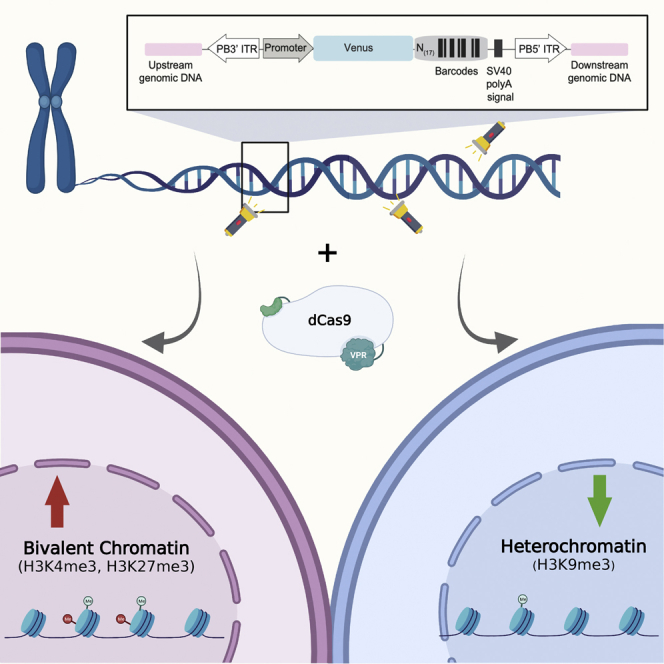

## Teaser

Wu et al. use a barcoded reporter system to show that CRISPR-based activation of gene expression is effective in most contexts in stem cells and neurons, but the level of activation depends on basal gene expression and chromatin status. They demonstrate that these rules also apply at endogenous genes.

## Introduction

A central challenge in functional genomics is to regulate the expression of thousands of individual genes precisely and efficiently. CRISPR-based epigenetic modification systems have enabled high throughput, targeted manipulation of epigenetic states, allowing studies of both the loss and gain of gene function. These techniques use a catalytically inactive Cas9 protein as a sequence-specific, DNA-binding moiety that recruits transcriptional activation (VPR, VP64, and SAM^1^^,^^2^^,^^3^) or repression (KRAB^3^) domains or chromatin-modifying proteins (p300, LSD1, and EZH2^4^^,^^5^^,^^6^) to activate or inhibit gene or regulatory-element function. Although these techniques have been broadly used in the gene-regulation field, it remains challenging to predict the efficiency of CRISPR-mediated activation and repression at a particular genomic locus.

To assess the efficiency of CRISPR activation (CRISPRa) in different genomic contexts in a high-throughput manner, we integrated a minimal, barcoded reporter gene at thousands of sites across the genome of a human induced pluripotent stem cell (iPSC) line that can be induced to efficiently differentiate to neurons. Chromatin context clearly has a massive impact on the expression level of reporter genes, depending on their genomic integration site.^7^ Hence, the cellular state change from iPSCs to neurons provides us with an ideal platform to assess how genomic context and basal gene expression influence CRISPRa efficacy. Here, two types of CRISPRa constructs were tested with dead Cas9 (dCas9) fused to the transcription activator VPR or the histone acetyltransferase p300. Surprisingly, they behave very differently in pluripotent stem cells both for barcoded reporter genes as well as endogenous genes. dCas9-VPR was able to activate most barcoded reporter genes, independent of chromatin status, whereas dCas9-p300 cannot. We assessed the basal expression of endogenous and integrated reporter genes, as well as the ability of dCas9-VPR to activate the integrated reporters across thousands of different chromatin contexts in both iPSCs and differentiated neurons. We found that the dCas9-VPR outcome was highly dependent on basal expression level. Interestingly, the investigation of additional chromatin features affecting CRISPRa outcome highlights bivalent genes as being particularly sensitive to dCas9-VPR, highlighting the potential of using CRISPRa for manipulating stem cell differentiation in the future.^8^^,^^9^ Finally, we tested whether these rules can be applied to endogenous loci using a parallel single-cell-based CRISPRa assay. As expected, all of the tested bivalent genes can be strongly and robustly activated, but H3K9me3-marked regions are less responsive to CRISPRa. We also analyzed the absolute activation levels using our single-cell data and demonstrated that CRISPRa could elicit strong activation, which corresponds to the top 20% of endogenous gene expression levels.

## Results

### Creating a multiplexed barcoded human iPSC pool as a resource to study the context dependence of CRISPRa

**Figure 1:**
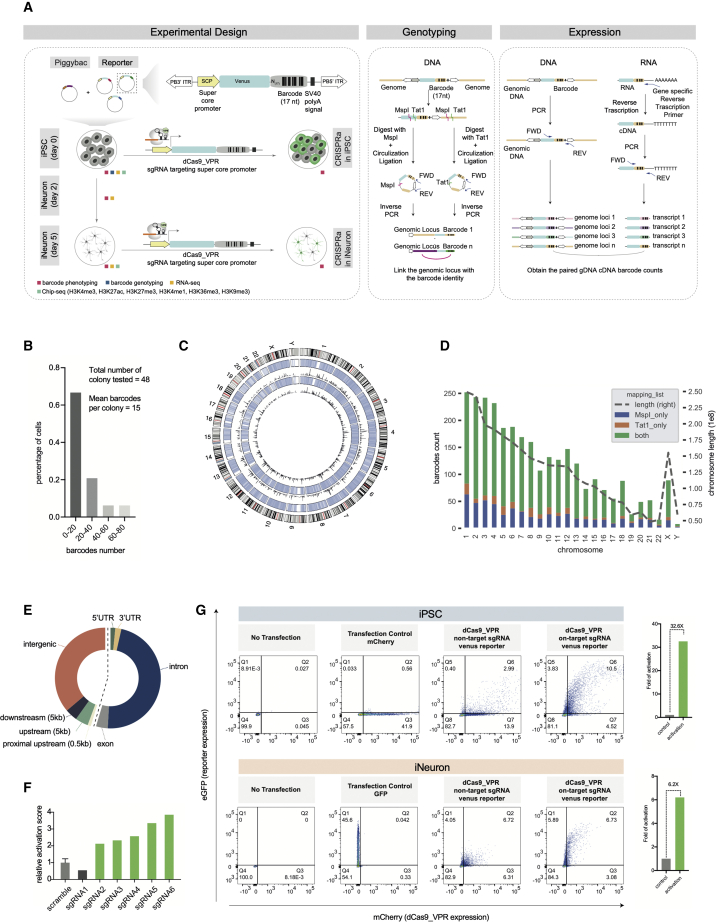
Parallel assessment of CRISPR sgRNA efficacy in different chromatin contexts (A) Overview of experimental design. (B) Barcode integration distribution at single-colony level. (C) Position of barcoded reporter insertions across chromosomes. Each integration is represented as a single semi-transparent blue line. The color intensity indicates the integration density for each chromosome. The outer blue circle represents integrations mapped with *Tat* I and the inner circle those mapped with *Msp* I. The black histogram shows the total mapped read counts. The outermost ring shows the human cytobands, with red highlighting the pericentromeric regions. (D) Mapping of 2,923 reporter insertions across chromosomes. Colored bars show the mapped barcode counts on each chromosome (left, y axis). The dashed line shows the total length of the chromosome (right, y axis). In total, 74.4% barcodes can be independently mapped by both enzymes, 19.6% can be mapped by *Msp* I only, and 5.9% mapped by *Tat* I only. (E) Distribution of reporters across genomic annotations. Intronic and intergenic insertions make up the two largest groups, with 47.3% and 36.2% of the barcodes, respectively. (F) FACS determined sgRNA efficacy in HEK-293T cells using the reporter vector. Three plasmids (dCas9-VPR, sgRNA, and Venus reporter) were co-transfected into HEK-293T cells. The fold change relative to the non-targeting control sgRNAs is shown in the histogram. Functional sgRNAs (2–6) are labeled in green. (G) FACS determined sgRNA efficacy in human iPSCs and induced iNeurons using the reporter vector. Transfection controls demonstrate a 41.9% or 45.6% transfection efficiency in hiPSCs and iNeurons, respectively. Relative to a mix of two non-targeting sgRNAs, a mix of the five targeting sgRNAs (sgRNA 2–6 on F) shows 32.6× and 6.2× activation of the Venus reporter in iPSCs and iNeurons, respectively. See also Figure S1.

To assay the effectiveness of CRISPR perturbations in different genomic contexts, we developed a multiplexed, integrated reporter assay and applied this to understanding CRISPRa efficacy. We employ a minimal reporter gene consisting of a synthetic core promoter and a Venus fluorescent protein with a randomized 17-nucleotide barcode in the 3′ UTR (Figure 1A). The synthetic core promoter contains four core promoter motifs. TATA box from the CMV IE1 core promoter, a composite initiator based on sequences from AdML and *Drosophila melanogaster* G retrotransposon core promoters, the motif ten element from the *Drosophila* Tollo core promoter, and the downstream promoter element from the *Drosophila* G core promoter.^10^^,^^11^ This was introduced across the genome of human iPSCs by co-transfecting the piggyBac transpose with a pool of barcoded reporters^12^ that integrate semi-randomly but with a preference for adenine and thymine (AT)-rich regions. Expression of thousands of barcodes can be assayed simultaneously by extracting genomic DNA and RNA from the pool of cells and performing high-throughput amplicon sequencing across the barcode in genomic DNA (gDNA) and RNA-derived complimentary DNA (cDNA). The ratio of cDNA/gDNA reads provides an accurate measure of expression of each barcode (Figure 1A).^13^ Importantly, the use of a reporter system means that we can use the same single guide RNA (sgRNA) at all loci, thus uncoupling any guide-specific effects from the effects of the chromatin environment.

In order to introduce a genome-wide epigenetic perturbation, we converted iPSCs to neurons and characterized the epigenetic changes that occur during this process. The iPSC line used for this experiment contained transgenes that allow a doxycycline-inducible expression of *NGN2*, which drives homogeneous, synchronous production of cortical neurons (iNeurons)^14^ (Figure S1A). Although this is unlikely to accurately reflect differentiation *in vivo*, it is still a highly reproducible model of a cell state change. Upon induction of the integrated *NGN2* transgene in our iPSC line with doxycycline, we observed a striking change in morphology over 5 days, consistent with this cell state transition.^14^ We analyzed the changes occurring at the level of the transcriptome by RNA sequencing (RNA-seq) and chromatin modifications by chromatin immunoprecipitation sequencing (ChIP-seq) (Figures 1A, S1B, S1C, and S1D). We measured six post-translational modifications of histones, comprising modifications marking promoters (H3K4me3^15^), poised enhancers (H3K4me1^16^) and active enhancers (H3K27ac^16^), transcribed regions (H3K36me3^17^), polycomb domains (H3K27me3), and constitutive heterochromatin (H3K9me3). This showed that there is a significant change in cell state during the first 5 days after *NGN2* induction, consistent with the acquisition of a neuronal fate (Figures S1E, S1F, and S1G). This provides an unusual opportunity to compare the expression of the same set of reporter integrations in the chromatin environments of iPSCs and iNeurons and subsequently assay the effect of chromatin context on CRISPR efficacy in distinctive cellular states.

By single-cell cloning and sequencing of barcodes, we demonstrated that each cell contained a mean of 15 reporter insertions (Figure 1B). We mapped integration sites of each reporter insertion using an inverse PCR strategy followed by high-throughput sequencing to link barcodes to a genomic location (Figure 1A). Most (74.4%) integration sites were independently mapped to the same site with two independent enzymes, indicating our mapping method is highly accurate (Figures 1C and 1D). Integrations were spread across the entire genome (Figures 1C and 1D) and covered most genomic annotations (Figure 1E), except pericentric regions (Figure 1C). As expected, we found piggyBac insertion is AT-region biased. The mean AT percentage is 62.4% (std = 8.8%) surrounding the 100-bp window of barcode insertion locus, compared with an average of 59.1% in the human genome. We identified 2,923 barcodes that could be confidently assigned to a single genomic location, which were used for all subsequent analyses (Table S1).

We next tested the CRISPRa efficiency of six sgRNAs targeting the super core promoter by co-transfecting each sgRNA individually into HEK-293T cells along with the Venus reporter vector and a plasmid expressing dCas9-VPR and an mCherry reporter gene. Compared with a mix of 2 non-targeting, scrambled sgRNAs, five of the six guides showed activation of the reporter ranging from 2.1- to 3.8-fold (Figure 1F). To minimize gRNA-specific effects, we performed all subsequent experiments with a mix of these five guides (sgRNA 2–6) and a mix of two scrambled guides as a control. This set of sgRNAs was able to robustly activate the expression of the reporter in both iPSCs (32.6× activation) and iNeurons (6.2× activation) (Figure 1G).

### Differentiation of stem cells to neurons dramatically changes the chromatin landscape and barcoded gene expression

**Figure 2:**
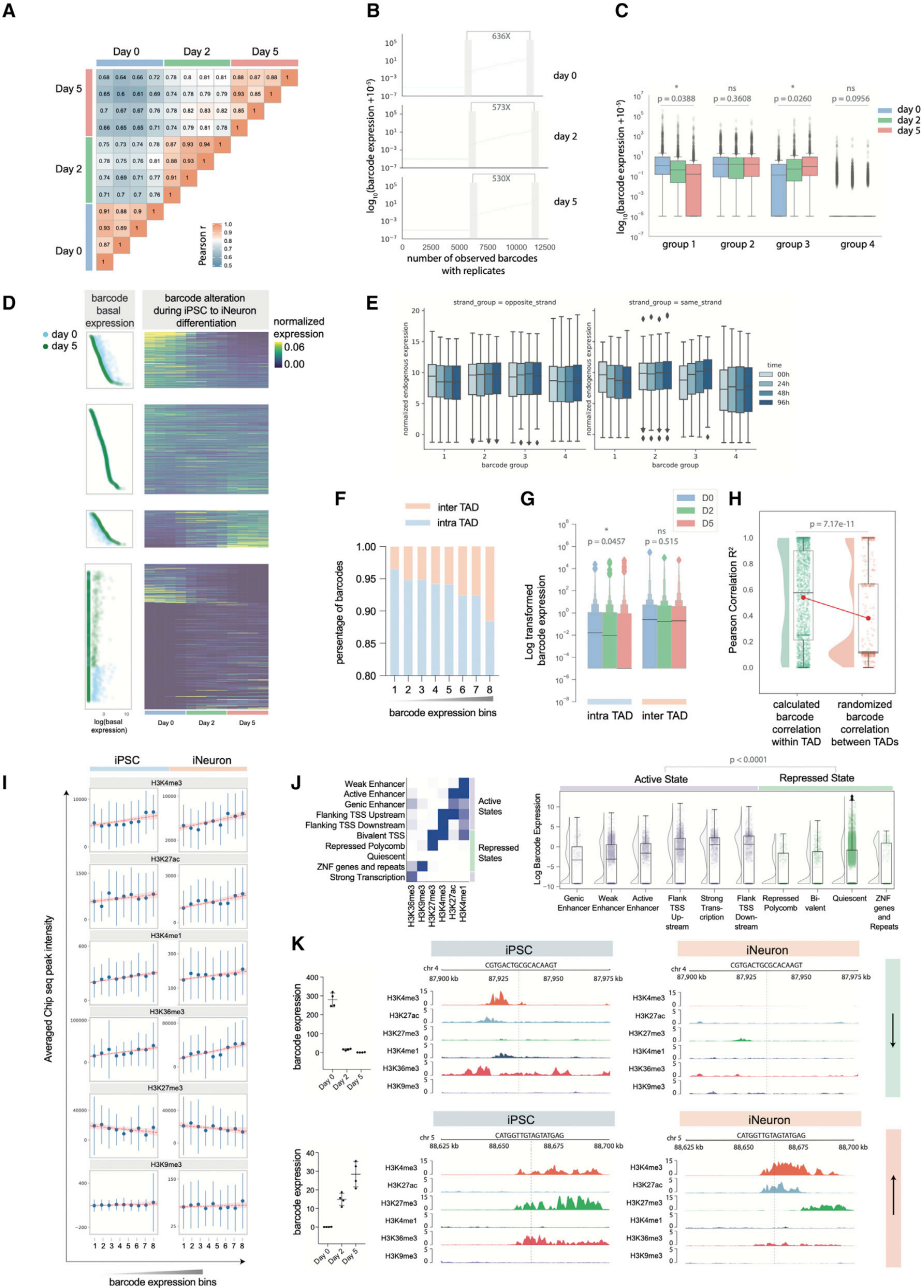
Reporter expression changes during iPSC to iNeuron differentiation (A) Reporter expression correlation matrix with all replicates. The heatmap shows the Pearson correlation of four biological repeats, using those reporters expressed between the 10th and 90th percentiles. (B) Basal expression distribution at days 0, 2, and 5 of iNeuron differentiation. In order to plot non-expressed barcodes, we added 0.00001 (<1% of the lowest-expressed barcodes). The fold change of barcode expression is between the mean of the top 10% and bottom 10% of expressed reporters within each time point. (C) Boxplot for barcode expression within four groups based on changes from iPSC to iNeuron. Boxplot shows the median, the first, and third quartile. Whiskers shows the 1.5 interquartile range. Group1: turned off (one-way paired ANOVA). Group2: constitutively on (one-way paired ANOVA). Group3: turned on (one-way paired ANOVA). Group 4: constitutively silenced (one-way paired ANOVA). The loliplot on the left panel show the log-transformed day 0 and 5 median expression level. (D) Barcode expression dynamics across iNeuron differentiation. Left panel: loliplot showing the log-transformed reporter basal expression with days 0 and 5 median barcode expressions. Right panel: heatmap shows reporter expression during the iPSC to iNeuron differentiation. (E) Normalized endogenous gene expression change (4 time points in total, 3 biological replicates for each) for the nearest gene to the reporter insertion. Plots are grouped by whether the reporter is on the same strand or a different strand from the endogenous gene. Boxplot shows the median, the first, and third quartile. Whiskers shows the 1.5 interquartile range. (F) Distribution of reporter integration within TADs (intraTAD) and between TADs (interTAD) across 8 ranked expression bins with both D0 (iPSC) and D5 (iNeuron) data. (G) Barcode expression changes during differentiation within intraTAD and interTAD groups. One-way ANOVA, intraTAD p = 0.515, interTAD p = 0.0457. (H) Pearson correlation coefficient of reporter expression within a TAD and randomly sampled barcodes between different TADs. (Welch’s t test, p = 7.17e−11) (I) The sum of ChIP-seq signals in a window 5-kb up- and downstream of reporter insertion sites. IntraTAD barcodes are divided into 8 expression bins (1 is undetectably expressed, 2–8 are low-high expression) and the mean ChIP-seq signal is shown (error bars show standard deviation). The lines show a linear regression model (shading indicates confidence interval). (J) Left panel: heatmap of emission parameters of the ChromHMM model. Right panel: raincloud plot showing the log-transformed barcode expression in each ChromHMM-defined state. The reporter expression was extracted from both iPSC to iNeuron differentiation experiments (n = 4 for both iPSC and iNeuron groups) and the iPSC and iNeuron dCas9-VPR activation experiment (n = 2 for iPSC and n = 4 for iNeuron). The independently observed barcode number for each chromatin state: generic enhancer = 252, weak enhancer = 3,622, active enhancer = 1,336, flank TSS upstream = 2,268, strong transcription = 1,296, flank TSS downstream = 1,126, repressed-polycomb = 326, bivalent = 702, quiescent = 28,822, and ZNF genes and repeats = 248. The reporter expression in active chromatin states is significantly different from that in repressed states (Mann-Whitney, p < 0.0001). (K) Two examples of ChIP-seq signals during differentiation, either turned off (top panel, chromosome 4: 87934421, Kruskal-Wallace test, p < 0.0001, n = 4 for each time point) or turned on (bottom panel, chromosome 5 : 88664976, Kruskal-Wallace test, p < 0.0001, n = 4 for each time point). See also Figure S2.

We next analyzed the barcoded reporter gene expression during a cell state transition to assess how changes in chromatin state influence the basal expression level of the reporters. Four biological replicates were analyzed at days 0, 2, and 5, which showed high concordance within time points (median R = 0.88 among replicates) (Figure 2A). As expected, reporter expression depended strongly on the genomic integration site, and 48.7% of barcodes showed an undetectable level of expression. When comparing the mean of the 10% lowest detectable reporters to the 10% highest expressed, we observed a 530-fold (iNeuron) to 636-fold (iPSC) variation in expression (Figure 2B). There were also substantial changes in reporter expression during iNeuron formation, and we used this to classify barcodes into four groups: turned off (group 1), constitutively on (group 2), turned on (group 3), or constitutively off (group 4) (Figures 2C and 2D). The average expression level of endogenous genes 5-kb up- or downstream of the insertion site showed a similar trend to the reporter expression (Figure 2E). Similar effects were observed at different window sizes ranging from 1 to 100 kb (Figure S2A) but were lower in magnitude as the distance increased. In order to perform this analysis, we analyzed endogenous genes within a window (1, 2, 5, 10, 50, and 100 kb) around the reporter integration site. We then separated the endogenous genes into two categories, depending on whether they were transcribed from the same or different strand as the barcoded reporter, and plotted the endogenous gene expression based on the barcode cluster groups and their strand groups. This demonstrates that the reporter integrations assay the chromatin and transcriptional enhancer context surrounding their integration site and respond to the changes in this state that occur during this cell fate transition. It also identifies a set of insertion sites that could be candidates for regulating transgene expression, either to maintain constitutive expression (group 2, “safe harbor” sites) or confine expression to iPSCs (group 1) or iNeurons (group 3) (Table S1). Selected candidate safe harbor loci are listed in Table S1 using criteria described in method details.

We next analyzed the chromatin features that could drive basal reporter expression, focusing on the post-translational modifications of histones and the higher-order chromatin folding assayed by chromatin conformation capture (HiC). A HiC dataset from human embryonic stem cells was used to segment the genome into topologically associated domains (TADs),^18^ and reporter insertions were classified as within (intraTAD) or outside (interTAD) TADs (Figures 2F and 2G). By binning reporter expression into 8 groups, we found that interTAD regions were enriched in highly expressed reporters that were constitutively active in iPSCs and iNeurons (Figures 2F and 2G). In contrast, reporters integrated within a TAD had a lower basal expression and in general were significantly repressed during iNeuron formation (one-way ANOVA, p = 0.04) (Figure 2G). Consistent with previous results, this suggests that intraTAD regions generally contain chromatin states subject to cell-type-specific regulation, whereas interTAD regions contain constitutively expressed housekeeping genes.^19^ It has been postulated that TADs demarcate chromatin domains that contain co-regulated genes.^20^^,^^21^ In agreement with this, we showed that during transition from iPSCs to iNeurons, the correlation (Pearson R^2^) of reporter expression within a TAD was significantly higher than the correlation observed with pairs of reporters in different TADs (Figure 2H) (p = 7.17e−11, Welch’s t test).

Post-translational modifications of chromatin have been correlated with changes in gene expression.^22^ However, such analyses are often confounded by differences in basal promoter architecture and post-transcriptional regulation of RNA levels. Our reporter system uses a consistent core promoter and regulatory elements, thus removing these variables. We binned the reporter integrations into 8 bins according to the basal expression level in iPSCs or iNeurons and looked for correlation with the level of different chromatin modifications across a 10-kb window upstream and downstream of the reporter insertion site. This showed that, independently of the cell state, the level of H3K4me3, H3K4me1, H3K36me3, and H3K27ac in the region surrounding the insertion was positively correlated with reporter expression, consistent with their role in active gene expression. Conversely, the polycomb marker H3K27me3 was inversely correlated with reporter expression, whereas levels of the constitutive heterochromatin marker H3K9me3 were independent of reporter expression (Figure 2I).

Combinations of different chromatin modifications can delineate a more refined set of chromatin states using a hidden Markov model (ChromHMM).^23^ We therefore trained ChromHMM using six histone modifications to define ten chromatin states (Figure 2J). These consisted of six active states, three repressive states, and a quiescent state devoid of any chromatin modifications.^24^ Reporters landing in active regions within enhancers or near active transcriptional start sites had a much higher expression than those integrating within repressed domains (Mann-Whitney, p < 0.0001) (Figure 2J). This demonstrates that the chromatin environment has a strong effect that at least partially predicts the basal expression of the reporters. In specific examples, chromatin state changes during iNeuron formation can also explain the changes in reporter expression that we observed (Figure 2K), such as the transition from an active to quiescent chromatin environment, or bivalent to active state.

### dCas9-VPR and dCas9-p300 exhibit different activation efficiencies in pluripotent stem cells

**Figure 3:**
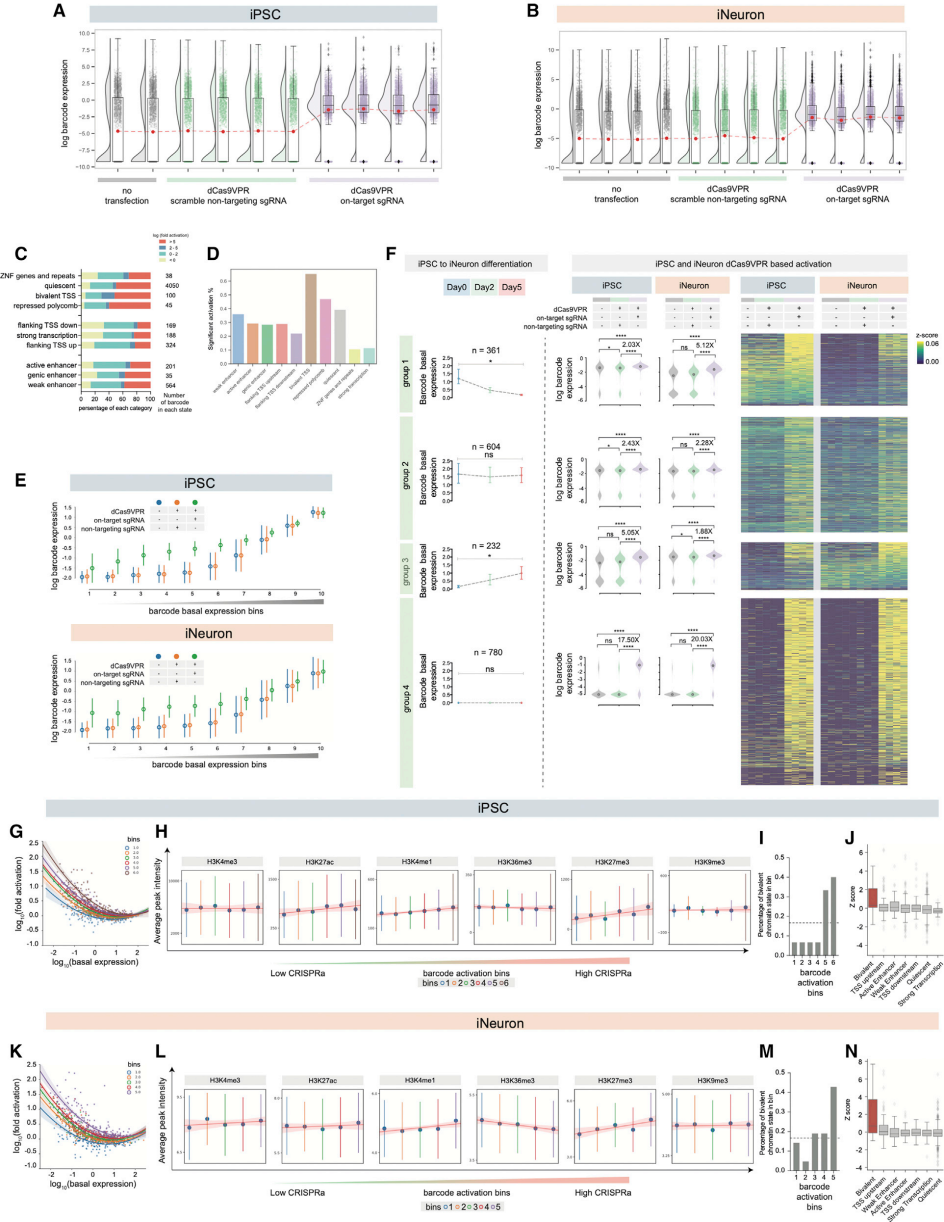
dCas9-VPR-mediated CRISPRa efficacy in iPSCs and iNeurons (A) dCas9-VPR-mediated reporter activation in iPSCs. Boxplot shows the median, the first, and third quartile, and the red dot shows the mean (Wilcoxon matched-pairs signed rank test between control sgRNA group and dCas9-VPR group, p < 0.0001; Wilcoxon matched-pairs signed rank test between no transfection group and dCas9-VPR group, p < 0.0001; Wilcoxon matched-pairs signed rank test between control sgRNA group and no transfection group, p = 0.0005). (B) dCas9-VPR-mediated reporter activation in iNeurons. Boxplot shows the median and the first and third quartile, and the red dot shows the mean (Wilcoxon matched-pairs signed rank test between control sgRNA group and dCas9-VPR group, p < 0.0001; Wilcoxon matched-pairs signed rank test between no transfection group and dCas9-VPR group, p < 0.0001; Wilcoxon matched-pairs signed rank test between control sgRNA group and no transfection group, p = 0.0895). (C) Log-transformed fold activation by CRISPRa across ChromHMM states in both iPSCs and iNeurons. (D) Proportion of cells with significant activation across ChromHMM states in iPSCs. (E) Reporter expression across different basal expression bins in iPSCs (top, n = 197 or 198 in each bin) and iNeurons (bottom, n = 197 or 198 in each bin). (F) Left panel: basal reporter expression changes during iPSC to iNeuron differentiation across 3 time points grouped into 4 groups. Median with 95% confidence interval is illustrated. Group 1: turned off (one-way paired ANOVA, p = 0.0388). Group 2: constitutively active (one-way paired ANOVA, p = 0.3608). Group 3: turned on (one-way paired ANOVA, p = 0.0260). Group 4: constitutively silenced (one-way paired ANOVA, p = 0.0956). Right panel: CRISPR activation efficacy across the 4 groups at both iPSC and iNeuron stages. Violin plot shows the data distribution and the median. Fold changes of means and significance are shown (paired t test, ns: non-significant, ^∗^ p < 0.05, ^∗∗^ p < 0.01, ^∗∗∗^ p < 0.001). Heatmap shows reporter activation in the 4 groups at iPSC and iNeuron stages. **(**G and K) The relationship between barcode basal expression and fold activation by CRISPRa. Activation bin 1 contains the least-activated reporters and bin 6 the most activated. The colored lines show the polynomial regression with an order of 2. Shading shows the confidence interval of the polynomial regression. In total, there are 200 barcodes in each iPSC bin and 211 barcodes in each iNeuron bin. (H and L) The average ChIP-seq peak intensity across all activation bins. ChIP peaks were considered within a 10-kb window up- and downstream of the reporter insertion site. The blue dots show the mean of the ChIP-seq signals in each bin (error bars show standard deviation). The red line shows the linear regression model fit (shading shows the confidence interval). Note that the bins in (G) are the same ones in (H), and those in (K) are the same ones in (L). (I and M) The number of reporter integrations within bivalent chromatin states across activation bins (for iPSCs, the observed number for each bin is 1,1,1,1,5,6; for iNeurons, the observed number for each bin is 3,1,4,4,9). Dotted line: theoretical number of bivalent status in each of the bins. (J and N) The *Z* score distribution of residuals to the exponential decay model for each chromatin status. See also Figures S3 and S4.

In order to assess the efficacy of CRISPRa across different chromatin states, we first transfected a dCas9-VPR^1^ construct along with a set of two non-targeting sgRNA plasmids—or the set of five sgRNAs targeting the reporter gene—into both iPSC and iNeuron cell types. In both cell states, reporter expression was globally increased only when dCas9-VPR was introduced together with the targeting sgRNA pool and not with the non-targeting sgRNAs (Figures 3A and 3B). As expected, similar results were obtained with dCas9-p300 in HEK-293T cells, (Figure S2B). Interestingly, when a similar experiment was performed using a dCas9-p300 construct in iPSCs, no significant global activation or activation of individual reporter insertions was observed (Figures S2C–S2E).

In order to further confirm this difference in dCas9-p300- and dCas9-VPR-mediated activation in pluripotent stem cells, we targeted two endogenous genes (Ascl1 and NeuroD1) using a human embryonic kidney cell line (HEK-293T), two iPSC cell lines (KOLF2-C1 and NGN2 OPTi-OX^14^), and one human embryonic stem (ES) cell line (H9) (Figures S2F and S2G). As a previous study showed,^5^ dCas9-p300 could dramatically activate endogenous gene expression in the differentiated cell line (HEK-293T), providing 2- to 4-fold higher activation than dCas9-VPR. Surprisingly, dCas9-p300 showed non-detectable or marginal gene activation in all three pluripotent stem cell lines.

### dCas9-VPR activation outcome depends on basal gene expression level and chromatin status

When using dCas9-VPR, CRISPRa-mediated activation was observed across all chromatin states (Figures 3C and 3D), and on average 58.1% of barcodes were activated more than 2-fold in iPSCs and 62.5% in iNeurons. Interestingly, reporter integrations in active chromatin environments were activated less frequently and to a lower extent than those within repressive chromatin states. We reasoned that basal expression could be a determinant of CRISPRa efficacy, so we binned reporter integrations into 10 bins according to their basal expression levels and analyzed the level of activation (Figures 3E and S3). This demonstrated that reporter integrations with lower basal expression were generally activated more strongly than those with high basal expression, which could not be hyperactivated by dCas9-VPR. When we grouped reporter insertions according to their expression patterns during iNeuron conversion (groups 1–4, Figures 2D and 3F), we similarly observed that the constitutively low set (group 4) was activated to a larger extent than the constitutively high group (group 2). Interestingly, those reporters that were turned off (group 1) or turned on (group 3) during iNeuron formation were activated more effectively in the cell type in which they had a low basal expression (Figure 3F). This demonstrates that even with the same reporter insertion, CRISPRa efficacy is strongly influenced by cellular state and basal gene expression level.

We next analyzed whether the basal expression level was sufficient to predict fold activation of reporter insertions. The log(basal expression) versus log(fold activation) showed a good fit to an exponential decay model at both iPSC and iNeuron stage (R^2^ = 0.59 for iPSCs and R^2^ = 0.52 for iNeurons) (Figures 3G, 3K, S3B, and S3C). Nevertheless, there was a degree of variability that was not explained by basal expression level (Figures 3G, 3K, S3B, and S3C). To investigate whether this could be dependent on particular chromatin states, we ranked the reporters into 6 groups that were susceptible to CRISPRa activation less (group 1) or more (group 6) than predicted from their basal expression level (Figures 3G, 3K, and S3A; method details). We found that reporter insertions that were activated more than expected were enriched in the enhancer markers H3K4me1 and H3K27ac and the polycomb marker H3K27me3 (Figures 3H and 3L). By analyzing chromHMM states, we found that the bivalent chromatin state was strikingly enriched in the groups that responded more strongly than expected to CRISPRa at both the iPSC and iNeuron stage (Figures 3I and 3M). Reinforcing this result, bivalent chromatin showed a significantly higher deviation from the exponential decay model when compared with all other chromHMM states (Figures 3J and 3N, one-way ANOVA for iPSCs, p < 0.0001; for iNeuron, p < 0.0001). This is consistent with the poised nature of bivalent chromatin, whereby an activating signal can set up a positive feedback loop to reinforce robust transcription.^25^^,^^26^ These results were further confirmed by analysis of individual examples of reporter insertions that were consistent with the overall trends observed here (Figure S4).

### Single-cell-based CRISPR activation of endogenous genes confirms chromatin-dependent effect

To demonstrate that dCas9-VPR-based activation of endogenous genes follows similar rules to the reporters, we performed a CRISPRa activation experiment targeting 96 genes across 10 different chromatin states with a single-cell transcriptomic readout.

**Figure 4:**
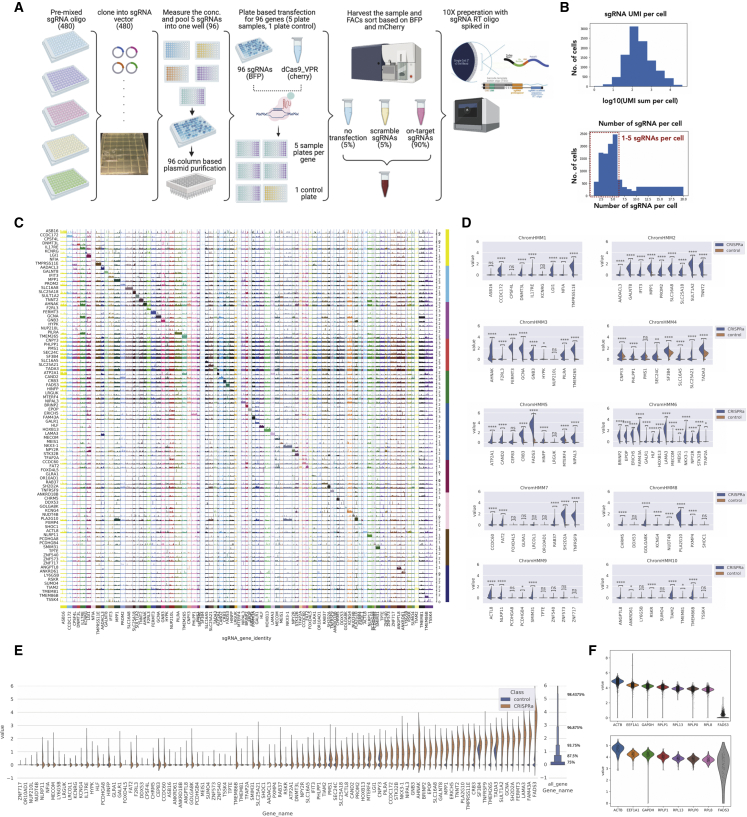
CRISPR activation screen of endogenous genes with a single-cell transcriptomic readout (A) Overview of the experimental design for the arrayed single-cell CRISPRa experiment. (B) Distribution of sgRNA UMI counts and number of unique sgRNAs per cell. (C) Track plot for each CRISPRa perturbation. The gene expression level is represented by height and the horizontal axis shows individual cells grouped by perturbation. (D) The raw normalized single-cell expression level with CRISPRa on-target activation (blue violin) and CRISPRa scramble control (orange violin) for all 10 ChromHMM status (Welch's t test independent samples with Bonferroni correction, ns: 5.00e−02 < p ≤ 1.00e+00, ^∗^: 1.00e−02 < p ≤ 5.00e−02, ^∗∗^: 1.00e−03 < p ≤ 1.00e−02, ^∗∗∗^: 1.00e−04 < p ≤ 1.00e−03, ^∗∗∗∗^: p ≤ 1.00e−04). (E) The comparison between the top-25% expressed endogenous genes (right panel) and the CRISPR activation levels (left panel). (F) Comparison of CRISPR activation levels per cell for FADS3 on-target (bottom panel) and scramble-target cells (top panel), with the top 7 expressed endogenous gene levels. See also Figure S5 and Data S3 and S4.

We first selected a group of genes across different basal expression levels by calculating the first, second, and the third quartiles of the gene expression for each ChromHMM chromatin state and extracted 20 genes across those values. Second, we manually confirmed that the chromatin profile of each gene corresponded to the assigned chromHMM state. Finally, we selected 9 genes for each chromatin state (3 genes for each quartile) and used CRISPick (https://portals.broadinstitute.org/gppx/crispick/public) to design 5 CRISPRa sgRNAs. To enrich for bivalent genes to test whether these could be activated and lead to any cell state changes in stem cells, we included an additional 6 genes that are bivalent in the iPSC stage, which also produced an effect in the TFome study.^27^ In total, 96 genes were included in this arrayed, single-cell CRISPRa experiment and each gene was targeted by 5 sgRNAs (480 sgRNAs in total) (Figure S5A; Table S2). A mix of 5 sgRNAs per gene were co-transfected with dCas9-VPR and compared with negative controls consisting of a mix of scrambled sgRNAs and dCas9-VPR or non-transfected cells. We harvested cell pools 48 h post transfection and enriched for positively transfected cells by fluorescence-activated cell sorting (FACS). Cells were mixed together (no transfection: scramble sgRNA: on-target sgRNA = 5:5:90) and analyzed by single-cell RNA sequencing (scRNA-seq) with direct guide capture (Figures 4A and S5B).^28^^,^^29^ In total, 47,375 single-cell transcriptomes were generated, comprising around 400 cells per endogenous gene CRISPRa perturbation.

First, we used CellRanger to assign sgRNA identities to each individual cell (sgRNA unique molecular identifiers UMI/cell median 208, mean 1,033). Because 5 sgRNAs were pooled and transfected together for each CRISPRa experiment, we expected most cells to contain 0–5 sgRNAs. Indeed, we found 6.1% of cells contained 0 sgRNAs, 81.27% of cells contained 1–5 sgRNAs, while 12.63% cells contained more than 5 sgRNAs (Figure 4B). To confidently assign each cell with a CRISPRa perturbation identity, we included cells with 1–5 sgRNAs that contained only sgRNAs from the set of five used for each gene. We identified 47,375 cells with a mean of 301 cells per perturbation (first and third quantile 209–345), and most CRISPRa perturbations (97.9%) represented by more than 40 cells. As controls, there were 2,642 cells containing scrambled sgRNAs and 18,182 cells without any sgRNAs. We excluded CEP83 and LRCOL1 from downstream analysis due to low cell numbers (15 and 13 cells containing sgRNAs). ANKHD1-EIF4EBP3 mRNA is an infrequent but naturally occurring readthrough transcript of the neighboring ANKHD1 and EIF4EBP3 genes, and because the data analysis pipeline (CellRanger) does not map this as a protein-coding gene, we also excluded this gene. After excluding these three genes, all subsequent analyses were performed with the remaining 93 perturbations.

We analyzed the response produced by each perturbation on its endogenous target gene and found that the majority of genes can be specifically activated by CRISPRa (Figure 4C). Interestingly, we found that chromatin is one of the determining factors for CRISPR activation outcome (Figure 4D). All genes in active enhancers (ChromHMM2, labeled by H3K27ac and H3K4me1) and bivalent (ChromHMM6, labeled by H3K4me3 and H3K27me3) chromatin can be significantly activated, while only 44.4% of genes assigned in zinc finger (ZNF) genes and repeat chromatin (ChromHMM9, labeled by H3K9me3) and 55.5% of quiescent chromatin (ChromHMM7, without any chromatin modifications) can be significantly activated (Figure 4D). In contrast to some previous reports, we found CRISPRa could achieve strong activation levels corresponding to an equivalent level of expression to the top 25% of all expressed endogenous genes (Figure 4E).^30^ For example, FADS3 CRISPRa yields a gene expression level comparable to the highest 7 endogenous genes in the entire single-cell dataset, including highly expressed housekeeping genes (ACTB, EEF1A, and GAPDH) and ribosomal genes (RPLP1, RPL13, RPL0, and RPL8). The activation effect seems to be largely independent of the number and identify of guides present in the cell, especially once they exceed 2 (Data S3 and S4)

**Figure 5:**
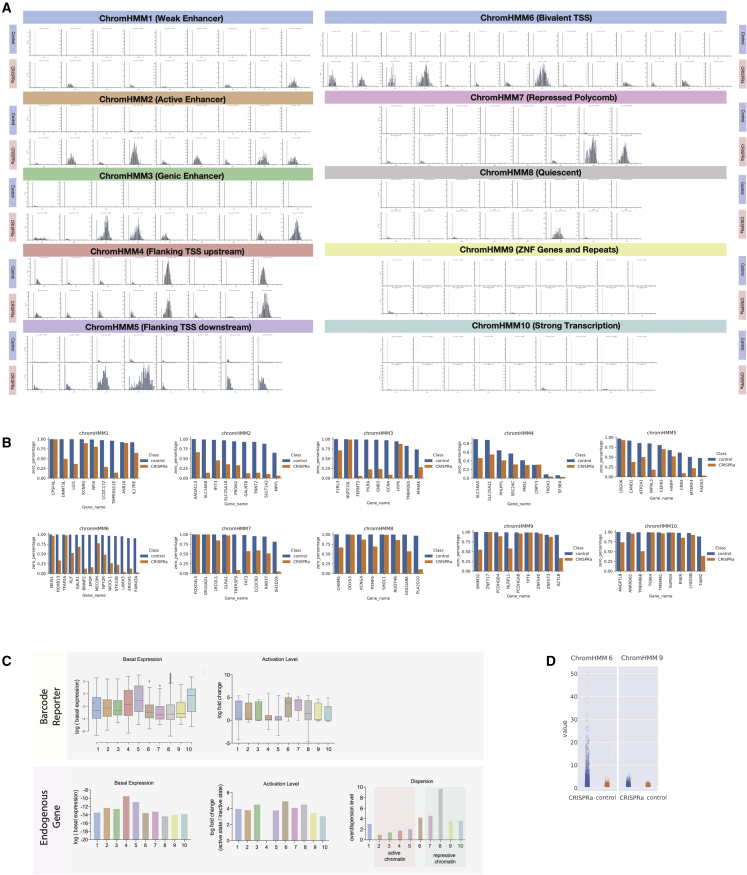
CRISPR activation is chromatin dependent at endogenous genes (A) Gene expression distribution plot with on-target sgRNAs (upper panels) and scrambled sgRNAs (lower panels) for each gene, grouped by chromHMM state. The x axis shows the binned expression level and y axis shows the density of cells in each bin. (B) The percentage of cells with 0 counts for each CRISPR perturbation grouped by chromHMM state. (C) Barcoded reporter (top panel) and endogenous gene (bottom panel) basal gene expression levels, activation levels, and model dispersion (measure of variance), grouped by chromHMM state (x axis). (D) The raw normalized gene expression levels for each cell with CRISPRa on-target guides (CRISPRa) or scrambled controls (control) in bivalent (ChromHMM6, H3K4me3 and H3K27me3) and ZNF-repressed (ChromHMM9 and H3K9me3) chromatin.

Next, instead of merging all cells containing the same CRISPRa perturbation, we examined the perturbation outcome in each individual cell (Figure 5A). Interestingly, we found that although on average CRISPR generated strong activation, not all cells could achieve high gene expression levels. In the control group, 90.1% cells (91.8%–99.6% for first and third quantiles) contain no detectable transcripts for a particular gene, while in the CRISPRa activated group, this decreased to 53% cells (22.1%–87.7% for first and third quantiles) (Figure 5B). This is likely for both technical and biological reasons. Technically, zeros could arise from mRNA not being captured and reverse transcribed, and stochastic sampling of cDNA in PCR or next-generation sequencing (NGS).^31^ Biologically, gene expression is inherently stochastic, and thus RNA transcripts are synthesized in discrete transcriptional bursts.^32^ Hence, it is important to model these zero data to understand the CRISPRa perturbation outcome. We assume that each cell could be in either of two latent states—“basal” or “active”—and then model the observed target gene UMI counts of each cell using a negative binomial distribution (see method details).

Interestingly, we found that both the basal gene expression level as well as the CRISPRa outcome at endogenous genes follow similar trends to our reporter experiment (Figure 5C). Specifically, flanking transcription start site (TSS) chromatins (upstream ChromHMM4 and downstream ChromHMM5) have the highest basal gene expression levels, and bivalent chromatins (ChromHMM6) achieve the strongest CRISPRa activation outcome. Furthermore, in general we observed lower variance in expression with active chromatin status (ChromHMM2, 3, 4, and 5, marked by H3K4me3, H3K27ac, and H3K4me1) with high variance in repressed or inactive chromatin states (ChromHMM7, 8, and 9, marked with H3K27me3 and no chromatin or H3K9me3 modifications). This indicated that CRISPRa resulted in more universal activation of gene expression in all cells within active chromatin, while activation is more stochastic in a repressed chromatin environment. To further demonstrate that bivalent chromatin (ChromHMM6) can achieve high levels of activation, whereas ZNF-repressed genes (ChromHMM9) can only be marginally activated, we analyzed raw normalized gene expression values in individual cells (Figure 5D). Although these two states show similar basal expression levels, bivalent genes were activated more strongly compared with the H3K9me3-repressed genes.

**Figure 6:**
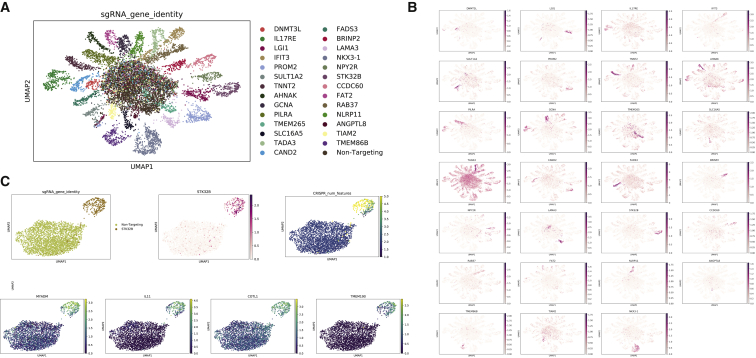
CRISPR activation of certain genes causes global changes in gene expression (A) UMAP projection of single-cell transcriptomic data colored by the CRISPRa target gene. (B) UMAP projection of single-cell transcriptomic data showing gene expression level of CRISPRa target genes. CRISPRa sgRNA targets are shown as titles, and CRISPRa targeted gene expression are shown with colors. (C) Gene expression changes upon CRISPR activation of STK32B. Guide assignment (top, left), STK32B expression (center, red), and number of sgRNAs per cell (top, right) are indicated, along with expression of HSC marker genes MYADM, IL11, COTL1, and TMEM190 (lower panels). See also Figure S5 and Data S1 and S2.

Finally, we analysed the transcriptome-wide response for each CRISPRa perturbation. In general, four classes of response were found within our dataset (Figure S5C; Data S1 and S2). Remarkably, in 54 (58.7%) cases, CRISPRa leads both to a significantly elevated gene expression as well as a shift in transcriptomic profile between the on-target and scramble sgRNA that is visible on the UMAP plot (Figures 6A, 6B, and S5C; Data S1 and S2). In 11 (11.9%) cases, CRISPRa appears to result in a change in the global transcriptome, yet we are not able to detect an increase in expression of the target gene, possibly due to the sensitivity of single-cell assay (Figure S5C). In a further 19 (20.6%) cases, CRISPRa causes significant activation of the target gene, but we do not observe any global transcriptomic perturbation (Figure S5C). In the final 8 (8.7%) cases, CRISPRa fails to cause activation of the target gene or a change in the transcriptome, which could be due to the sensitivity of the assay, chromatin status, or a non-functional sgRNA (Figure S5C).

Some of the CRISPR activated genes are transcription factors or chromatin regulators. To analyze whether activation of these genes could drive differentiation down particular cell lineages, we used the CellNet package to compare the differentially expressed genes with known markers of certain cell types (Figure S5D). We found that the activation of MEIS1 resulted in gene expression changes that partially overlapped with the profile of dendritic cells. However, activating GALR1 or STK32B caused changes that displayed similarities to the profile of hematopoietic stem cells (HSCs) (Figure S5D). We found that multiple HSC-related genes were activated in the STK32B CRISPRa cluster (Figure 6C). These included MYDAM, which is a hematopoietic-associated marker gene, IL11, which stimulates the proliferation of human hematopoietic CD34+ cells, COTL1, which maintains and regulates the homeostasis of HSCs, and TMEM190, which controls hematopoietic progenitor cell differentiation. Together, these data suggest that CRISPRa can drive high levels of gene activation, which is sufficient to drive cellular state changes and could be used for screening for factors that drive such changes.

We have now integrated ChromHMM data into our CRISPR design tool website (https://wge.stemcell.sanger.ac.uk/) to enable the selection of optimal guides for CRISPRa experiments.

## Discussion

The chromatin environment and genomic context play an important role in the transcriptional activity of integrated transgenes,^13^ but how this affects their ability to be perturbed by CRISPRa and across different cell types remains unclear. We set up a highly multiplexed barcoded reporter iPSC line, which allows simultaneous investigation of the expression of thousands of barcoded reporter genes during the iPSC to iNeuron transition. We characterize this cell state change at the level of the transcriptome and chromatin modifications and show that the reporters sample the chromatin environment in which they sit and the changes to this environment that occur during the formation of neurons. We also demonstrate that our barcoding technology has the potential to identify new safe harbor loci in an unbiased manner that can work across multiple cell types.

Using this system, we are able to uncouple the effect of guide RNA sequence and basal promoter from chromatin environment, and demonstrate that CRISPRa with dCas9-VPR^1^ and dCas9-p300^5^ is similarly effective in HEK-293 cells but behave very differently in pluripotent stem cells. It is perhaps surprising that dCas9-p300 is only able to cause marginal or no activation in hPSCs, but we propose the following possible explanations. First, overexpression of p300 could be lethal in pluripotent stem cells. However, we have titrated the amount of dCas9-p300 delivered and still observed no activation. Second, the pluripotent stem cell genome has a very different epigenetic state to other cell types and thus may respond differently to additional p300-mediated activation. Previous research has shown that chromatin is more permissive in pluripotent stem cells^33^^,^^34^^,^^35^ and that chromatin proteins are only loosely bound to chromatin, consistent with our observation of a reduced signal-to-noise ratio in our H3K27ac ChIP. Alternatively, the kinetics of addition or removal of the H3K27ac modification could be different in these cells.^36^^,^^37^ Finally, there could be a post-transcriptional regulation of p300 mRNA or protein levels or catalytic activity that prevents it from being able to activate transcription.

We next focused on dCas9-VPR and showed that it is effective in most chromatin contexts in both iPSC and iNeuron stages. The degree of activation is dependent on the basal expression level, with high expressing genes being difficult to activate further. While this is a general rule, chromatin state also has an impact, and bivalent genes are able to be activated more than would be expected. This is consistent with their biological role in bi-stable switching of key developmental genes and highlights that even developmentally repressed genes can be robustly activated by CRISPRa. Although allowing simultaneous quantitation of thousands of different integrations, our reporter system is biased in terms of the integration sites—meaning that we may not sample all possible genomic contexts—and uses an exogenous promoter, which does not reflect the natural situation. Therefore, we further examined whether these rules applied at endogenous genes using CRISPRa coupled to single-cell transcriptomics and found similar chromatin dependence of activation. Bivalent genes can be strongly and universally activated while H3K9me3 repressed genes are less responsive to the CRISPRa machinery. Single-cell analysis further showed that while most genes can be activated by CRISPRa, not every cell responded to the same extent, especially for H3K9me3-repressed genes, which showed a greater variation in response. We also found that dCas9-VPR could achieve high overexpression levels, similar to the top-expressed endogenous housekeeping genes. This was sufficient to cause significant changes in cellular state and transcriptome in pluripotent stem cells and drive features partially reminiscent of differentiated cell types, including HSCs and dendritic cells.

Our data demonstrate, for the first time, that dCas9-VPR-mediated transcriptional activation is generally applicable across chromatin states and cell types, but that basal expression level and chromatin state can impact both the degree of activation and its variability. These features will be important in the design and analysis of CRISPRa screens and the use of these systems for disease modeling or therapeutic intervention. Given the plethora of different dCas9 epigenetic modifiers that have recently been developed, it will be interesting to analyze how chromatin context affects the efficacy of other transcriptional or chromatin-modifying domains and their usefulness in modulating the regulatory landscape of a cell.

### Limitations of the study

There are some technical limitations to our study in that we used piggyBac to insert barcodes into the genome, which has a somewhat non-random integration pattern biased toward AT-rich regions and thus means that we may not have sampled chromatin states evenly. Also, our validation was done with a single-cell methodology which, while powerful in terms of absolute quantification, is limited by the capture rate of transcripts in single cells. Importantly, we have only analyzed two cell types, iPSCs and iNeurons, and validated the reporter results at a set of 93 endogenous genes, and thus different rules could apply in other cell types or genes. However, given that the general principles are similar between the reporter system and endogenous genes and in both iPSCs and iNeurons, we believe that these principles will apply more broadly across other cell types and systems. We have also only analyzed dCas9-VPR in detail, and thus the results could differ with other epigenetic or transcriptional modifiers, which would be of interest to study in the future.

## STAR★Methods

### Key resources table

**Table:** 

REAGENT or RESOURCE	SOURCE	IDENTIFIER
**Antibodies**		

Histone H3K4me3	Diagenode	Cat# C15410003; RRID:AB_2924768
Histone H3K27ac	Diagenode	Cat# C15410196; RRID:AB_2637079
Histone H3K4me1	Active Motif	61633; RRID:AB_2793712
Histone H3K27me3	Diagenode	C15410069; RRID:AB_2814977
Histone H3K36me3	Abcam	Ab9050; RRID:AB_2814977
Histone H3K9me3	Abcam	Ab8898; RRID:AB_306848

**Bacterial and virus strains**		

DH10B	NEB	C3019H
Biological samples		

**Chemicals, peptides, and recombinant proteins**		

ROCK inhibitor	Stem Cell Technologies	Y-27632
N-2 Supplement	Cell Therapy Systems	A1370701
B-27™ Supplement	Gibco	17504044
Recombinant Human NT-3 Protein	R&D systems	267-N3-025
Recombinant Human BDNF Protein	R&D systems	248-BD-005

**Critical commercial assays**		

RNA extraction (RNaeasy kit)	Qiagen	74004
reverse transcription (QuantiTect Reverse Transcription Kit)	Qiagen	205311
qPCR (SYBR Green Real-TimePCR Master Mixes)	Invitrogen	4309155
TransIT 2020	Mirus Bio	MIR 5404
TransIT LT1	Mirus Bio	MIR 2300
Bob_Ngn2 iNeuron electroporation	Lonza	P4 Primary Cell 4D-Nucleofector™ kit

**Deposited data**		

Raw BOB_Ngn2 RNAseq during iNeuron differentiation	This paper	EGA: EGAS00001004238
Bob_Ngn2 iPSC and iNeuron ChIPseq data	This paper	EGA: EGAS00001003165
Single cell CRISPR activation data	This paper	EGA: EGAS00001005528
Code for analysis of barcode and chromatin data	This paper	Zenodo: https://doi.org/10.5281/zenodo.7650425

**Experimental models: Cell lines**		

BOB_Ngn2 induced pluripotent stem cell line	Provided by Mark Kotter (University of Cambridge^14^)	n/a
KOLF2_C1 induced pluripotent stem cell line https://hpscreg.eu/cell-line/WTSIi018-B-1	Sanger HipSci	https://www.hipsci.org/lines/#/lines/HPSI0114i-kolf_2
HEK-293	ATCC	CRL 3216
Human H1 embryonic stem cell line	Provided by Antonio Vidal Puig (University of Cambridge)	n/a

**Oligonucleotides**		

caccgCGGGAGAAAGGAACGGGAGGgt (Ascl1_sgRNAa_FWD)	IDT	n/a
taaaacCCTCCCGTTCCTTTCTCCCGc (Ascl1_sgRNAa_REV)	IDT	n/a
caccgGGTCCGCGGAGTCTCTAACgt (NeuroD1_sgRNAd_FWD)	IDT	n/a
taaaacGTTAGAGACTCCGCGGACCc (NeuroD1_sgRNAd_REV)	IDT	n/a
CACCGCTGAAAAAGGAAGGAGTTGAGT (scramble_sgRNAa_FWD)	IDT	n/a
TAAAACTCAACTCCTTCCTTTTTCAGC (scramble_sgRNAa_REV)	IDT	n/a
CACCGAAGATGAAAGGAAAGGCGTTGT (scramble_sgRNAb_FWD)	IDT	n/a
TAAAACTCAACTCCTTCCTTTTTCAGC (scramble_sgRNAb_REV)	IDT	n/a
CGCGGCCAACAAGAAGATG (human Ascl1 FWD primer)	IDT	n/a
CGACGAGTAGGATGAGACCG (human Ascl1 REV primer)	IDT	n/a
GGATGACGATCAAAAGCCCAA (human NeuroD1 FWD primer)	IDT	n/a
GCGTCTTAGAATAGCAAGGCA (human NeuroD1 REV primer)	IDT	n/a
GTGACTGGAGTTCAGACGTGTGCTCT TCCGATCTCTGCATTCTAGTTGTGGTT TGTCC (Inverse PCR primer FWD)	IDT	n/a
ACACTCTTTCCCTACACGACGCTCTTCCG ATCTACGCAGACTATCTTTCTAGGGTTAA (Inverse PCR primer REV)	IDT	n/a
AATGATACGGCGACCACCGAGATCTACA CNNNNNNNNACACTCTTTCCCTACACGA CGCTCTTCCGATCT (NGS indexing primer FWD)	IDT	n/a
CAAGCAGAAGACGGCATACGAGATNNNNN NNNGTGACTGGAGTTCAGACGTGTGCTCTT CCGATCT (NGS indexing primer REV)	IDT	n/a
CTGCATTCTAGTTGTGGTTTGTCC (gene specificRT primer)	IDT	n/a
ACACTCTTTCCCTACACGACGCTCTTCCGAT CTAGCAAAGACCCCAACGAGA(Reporter first step PCR FWD)	IDT	n/a
GTGACTGGAGTTCAGACGTGTGCTCTTCCG ATCTTGGTTTGTCCAAACTCATCAATGTATC (Reporter first step PCR REV)	IDT	n/a

**Recombinant DNA**		

pGL4.23+SCP+SynIntron+Venus vector	Stein Aert’s lab^10^	n/a
sgRNAs backbone	Addgene	67990

**Software and algorithms**		

ChromHMM	Jason Ernst et al.	http://compbio.mit.edu/ChromHMM/
CellRanger	10x genomics	https://support.10xgenomics.com/single-cell-gene-expression/software/overview/welcome

**Other**		

Resource website containing ChromHMM data projected onto sgRNAs	This paper	https://wge.stemcell.sanger.ac.uk/

### Resource availability

#### Lead contact

Further information and requests for resources and reagents should be directed to and will be fulfilled by the Lead Contact, Dr. Andrew Bassett (ab42@sanger.ac.uk).

#### Materials availability

All requests for resources and reagents including plasmids and cell lines should be directed to the lead contact. All reagents will be made available on request after completion of a Material Transfer Agreement. There are restrictions on the availability of the OPTI-OX hiPSC line due to recent commercialisation and consents of the original donor.

### Experimental model and subject details

Cell lines HEK-293T cell lines were purchased from ATCC and cultured in Dulbecco’s Modified Eagle Medium (DMEM) supplemented with 10% fetal bovine serum (FBS) at 37°C and 5% CO_2_.

The corrected A1ATD line (BOB, https://hpscreg.eu/cell-line/CAMi014-A) and KOLF_2_C1 (https://hpscreg.eu/cell-line/WTSIi018-B-1) were male hiPSC lines generated as part of Cambridgeshire 1 NRES REC Reference 09/H0304/77, Hertfordshire NRES REC Reference 08/H0311/201, London Fulham REC Reference 14/LO/0345 and 15/LO/1126), HMDMC 14/013. The BOB_NGN2 OPTi-OX hiPSCs line was a genome edited derivative of the above corrected A1ATD BOB hiPSC line and was kindly provided by Mark Kotter (University of Cambridge^14^).

All hPSC cells were cultured in Essential 8™ Medium (Gibco™) on vitronectin (Gibco™, 100x) at 37°C and 5% CO_2_. Identity was recently confirmed by whole genome sequencing.

### Method details

#### Barcoded reporter plasmid library construction

The pGL4.23+SCP+SynIntron+Venus vector was kindly provided by Stein Aert’s lab.^10^ It contains super core promoter (SCP),^38^ synthetic-intron, Venus fluorescent protein and SV40 polyA signal. In order to integrate reporters using piggybac transposase, we first cloned the entire cassette into a piggybac vector using *Pac* I and *Pme* I restriction enzymes, forward primer ACGTTAATTAAGTACTTATATAAGGGGGTGGGGGCG and reverse primer ACGGTTTAAACAAAAAACCTCCCACACCTCCCC. Subsequently, to insert the 17bp barcode into the vector, we carried out an inverse PCR using the forward primer TAT**GGCGCGCC**TTACTTGTACAGCTCGTCCATGC and the reverse primer GTC**GGCGCGCC**GATCNNNNNNNNNNNNNNNNNGCTTCGAGCAGACATGATAAGATAC. Here, N stands for 25% of A,T,C,G at each base pair and GGCGCGCC is an *Asc* I restriction enzyme site. In order to prevent the synthesis bias with N contained base pairs, we independently synthesised 4 replicates of randomized barcode-containing reverse primer (IDT). Furthermore, to prevent PCR amplification bias, 96 independent PCR reactions (10μl in volume) were performed for each reverse primer replicate. Hence in total, we carried out 384 independent inverse PCR using KAPA HiFi HotStart ReadyMix (KAPA Bioscience) and pooled them together afterwards (PCR conditions: 56ºC annealing, 2.5 min extension and 27 cycles). The reactions were purified using a PCR purification kit (Zymo Research). In total 10 μg of the PCR product was digested with *Asc* I, purified and self-ligated. DNA ligation was performed at low concentration (2 ng/μl) in order to favour intramolecular interaction and ligation reactions were left at 16ºC overnight. Upon ligation using T4 ligase (NEB) and purification with Zymo purification kit, around 5μg of DNA was recovered. Subsequently, we electroporated 5 μg of barcode containing vectors into DH10B cells (NEB), recovered in 500 ml liquid cultures overnight and purified DNA using a maxiprep kit (Qiagen). In total, 1,812,160 unique barcodes were observed after high throughput sequencing using an Illumina miSEQ instrument.

#### Super core promoter targeting sgRNAs synthesis and functionality test in HEK-293T cells

In total, six sgRNAs were designed flanking the 81bp SCP region using NGG as PAM. The identity of the sgRNAs were listed in the table below. All sgRNAs were cloned into backbone (Addgene:67990) using Zhang lab protocol.^39^^,^^40^

**Table:** 

sgRNA oligo name	sgRNA oligo sequence	sgRNA name on Figure 1F
SCP_sgRNAa_FWD	CACCGCGAGTGTTCGATCGCGACTGGT	sgRNA3
SCP_sgRNAa_REV	TAAAACCAGTCGCGATCGAACACTCGC	
SCP_sgRNAb_REV	CACCGGAGCCGAGCAGACGTGCCTAGT	sgRNA4
SCP_sgRNAb_REV	TAAAACTAGGCACGTCTGCTCGGCTCC	
SCP_sgRNAc_FWD	CACCGGGTCCGTAGGCACGTCTGCTGT	sgRNA1
SCP_sgRNAc_REV	TAAAACAGCAGACGTGCCTACGGACCC	
SCP_sgRNAd_FWD	CACCGGTACTTATATAAGGGGGTGGGT	sgRNA6
SCP_sgRNAd_REV	TAAAACCCACCCCCTTATATAAGTACC	
SCP_sgRNAe_FWD	CACCGTAATTCGGGCCCCGGTCCGTGT	sgRNA5
SCP_sgRNAe_REV	TAAAACACGGACCGGGGCCCGAATTAC	
SCP_sgRNAf_FWD	CACCGGCAGACGTGCCTACGGACCGGT	sgRNA2
SCP_sgRNAf_REV	TAAAACCGGTCCGTAGGCACGTCTGCC	
scramble_sgRNAa_FWD	CACCGCTGAAAAAGGAAGGAGTTGAGT	Scramble sgRNA1
scramble_sgRNAa_REV	TAAAACTCAACTCCTTCCTTTTTCAGC	
scramble_sgRNAb_FWD	CACCGAAGATGAAAGGAAAGGCGTTGT	Scramble sgRNA2
scramble_sgRNAb_REV	TAAAACTCAACTCCTTCCTTTTTCAGC	

The functionality of individual sgRNAs was tested by transiently co-transfecting a mix of dCas9-VPR-cherry vector, sgRNA-BFP vector and SCP-Venus reporter vector into HEK-293T cells and subsequently detecting the Venus reporter expression levels by FACS. Cells were cultured in DMEM 4.5g/L glucose without L-Glutamine (Lonza) supplemented with 10% fetal bovine serum, 1% GlutaMAX (Life Technologies) and 1% Non-Essential Amino Acids Solution (Life Technologies) at 37 °C and 5% CO_2_. For transfection experiments, 10^5^ HEK-293T cells were plated into 12-well plates one day before transfection. In total, 1 μg of vector mix was transfected using 3 μl Transit 2020 (Mirus Bio) and 100 μl opti-MEM (Invitrogen). The ratio of sgRNA : dCas9-VPR : Venus-reporter = 1.5 : 1.5 : 1 (375ng sgRNA, 375ng dCas9-VPR and 250ng Venus-reporter) was used. Individual sgRNA activation was represented as fold change relative to the non-targeting control sgRNAs. This was calculated by multiplying the percentage of Venus-positive cells to the mean fluorescence intensity.

#### iPSC and iNeuron cell culture, transfection, and sgRNA functionality test

The pooled sgRNA experiments were carried out in BOB_NGN2 OPTi-OX hPSCs line as both iPSC cells and iNeuron cells using an equimolar mix of sgRNA 2,3,4,5 and 6 vectors.

iPSC cells (BOB_NGN2 OPTi-OX) were cultured in Essential 8™ Medium (Gibco™) on vitronectin (Gibco™, 100x) at 37 °C and 5% CO_2_ and transfected using reverse transfection. Firstly, 600 μl of Essential 8™ Medium with ROCK inhibitor (Stem Cell Technologies,Y-27632, 10 μM) was added into each well of a 6 well plate. Secondly, the vector mix (4 μg in total, dCas9-VPR: sgRNA is 2 : 1 with 2.6μg dCas9-VPR and 1.4 μg pooled sgRNAs), 12 μl of Transit-LT1 (Mirus Bio) and 400 μl of Opti-MEM were mixed and incubated at room temperature for 30 minutes. During the incubation, iPSCs were dissociated into single cells using accutase (Gibco™, 6 mL) at 37ºC for 4 minutes. An equal volume of media (6 mL) was added, cells were centrifuged at 300g for 3min, and washed once with culture media to thoroughly remove residual accutase. iPSCs were diluted to 500,000 cell/mL using Essential 8™ Medium with Rock inhibitor and 1 mL was added to 400μl of Transit-LT1 vector mix in each well.

For iNeuron induction and electroporation, iPSC were plated as single cells using Essential 8™ Medium with Rock inhibitor (Stem Cell Technologies, Y-27632, 10 μM) on vitronectin (Gibco™, 100x) for one day (Day 0). The iPSC culturing media was changed to M1 media at Day 1 and Day 2, consisting of DMEM/F-12 HEPES (Gibco™), N-2 Supplement (100x, Cell Therapy Systems), 2-Mercaptoethanol (50μM), GlutaMAX™ Supplement (Gibco™, 100x), MEM Non-Essential Amino Acids Solution (Gibco™,100x), 2-Mercaptoethanol (50 μM) and doxycycline (1 μg/mL). At Day 3, induced iNeurons were dissociated into single cells by incubating with accutase (Gibco™) at 37ºC for 4 minutes. These cells were washed with M1 media once to thoroughly get rid of residual of accutase. We electroporated cells using 5x10^5^ cells and 1μg plasmid DNA in 20 μl strips using the P4 Primary Cell 4D-Nucleofector™ kit (Lonza) with the program CA137 and achieved 45% delivery efficiency. After nucleofection, cells were immediately and gently plated into new 24-well plates coated with Geltrex™ (Gibco™). Each well contained warmed M2 media, constituting of Neurobasal™-A Medium (Gibco™), B-27™ Supplement (50X, Gibco™), 2-Mercaptoethanol (50 μM), Recombinant Human NT-3 Protein (10 ng/mL, R&D systems), Recombinant Human BDNF Protein (10 ng/mL, R&D systems), GlutaMAX™ Supplement (Gibco™, 100x), MEM Non-Essential Amino Acids Solution (Gibco™,100x), Rock inhibitor (Stem Cell Technologies, Y-27632, 10 μM) and doxycycline (1 μg/mL). At Day 4, Rock inhibitor free M2 media was replaced. Flow cytometry analyses were carried out at Day 5.

#### Characterization of human iPSC and iNeuron cell line (RNA-Seq and ChIP-Seq)

In order to characterize the iPSC and iNeurons (without integrated barcodes) we carried out polyA RNA-Seq at 4 time points with 3 replicates at each time point (0, 24, 48 and 96 hours post Ngn2 induction). Transcriptome libraries were generated with the Illumina TruSeq stranded RNAseq kit and all samples were sequenced using Illumina HiSeq with 40 million mapped reads on average for each sample. We performed ChIP-Seq using Tn5-based ChIPmentation protocol^41^ for 6 chromatin modifications. Antibody type, amount and cell number used in this study are listed in the table below. For ChIPmentation, 1 million iPSC or iNeuron cells were crosslinked and snap frozen. iPSC samples were sonicated using a Covaris E220 with 5% duty factor, 105w PIP, 200 CBP and 160s treatment time. iNeurons were sonicated using 10% duty factor, 140w PIP, 200 CBP and 120s treatment time. All the other steps followed the standard protocol.^41^ All ChIP-seq samples were sequenced using Illumina HiSeq with 50 million reads on average for each sample.

**Table:** 

Histone modification marks	Antibody source	Antibody amount used per ChIP
H3K4me3	Diagenode C1541003-50	0.5μl
H3K27ac	Diagenode C154101196	1μl
H3K4me1	Active Motif 61633	1μl
H3K27me3	Diagenode C15410069	1μl
H3K36me3	Abcam Ab9050	1μl
H3K9me3	Abcam Ab8898	1μl

#### Generation and characterization of barcoded reporter iPSC cell line

To integrate the barcoded reporters into the genome, we transfected iPSCs with a mix of barcoded reporter vector and Piggybac transposase. A mix of 5.25 μg barcoded library vector and 9.75 μg piggybac transposase was transfected into 3 million iPSCs using 45 μl of Transit-LT1. After 48 hours, we sorted Venus positive cells to enrich for positively transfected cells. To reduce the total number of reporter integrations, this complex pool of barcoded iPSCs were cultured and 20,000 cells were sorted into 6 well plates. In order to ensure complete loss of transient expression, cells were cultured whilst maintaining at least 100X coverage for one month before any downstream analysis. To characterize the barcode integration frequency, we sorted single cells into 96 well plates using FACS. Cells were cultured for 2 weeks and colonies lysed in squishing buffer (10 mM Tris-HCl, pH=8; 1 mM EDTA; 25 mM NaCl; 200 μg/ml Proteinase K). All samples were incubated at 65°C for 30 min and proteinase K was inactivated at 95°C for 2 min. Barcode integration was mapped as described below (Expression of reporters).

#### iPSC to iNeuron conversion and CRISPR activation

To monitor the reporter expression changes from iPSC to iNeuron, we differentiated iPSCs using the protocol above and collected 4 independent samples of 2 million cells at day 0, day 2 and day 5. For the CRISPR activation experiment in iPSC cells, we transfected a mix of 2.6 μg dCas9-VPR or dCas9-p300 and 1.4 μg pooled sgRNAs into one 6 well of iPSC (5 x 10^5^ cells). We used a pool of 5 targeting or 2 scrambled sgRNAs. We transfected 4 wells in 6 well format for each biological replicate. For CRISPR activation experiment in iNeuron cells, we electroporated 1 μg DNA (666 ng dCas9-VPR or dCas9-p300 and 333 ng pooled sgRNAs) into 5 x 10^5^ day 3 iNeurons four times for each biological replicate.

#### dCas9-p300 endogenous target activation experiment in iPSCs, human embryonic stem cells, and HEK-293T cells

Human iPSC cells (NGN2 OPTi-OX, KOLF2_C1) and human embryonic stem cell (H9) were cultured in Essential 8™ Medium (Gibco™) on vitronectin (Gibco™, 100x) at 37 °C and 5% CO_2_ and transfected using reverse transfection. Firstly, 600 μl of Essential 8™ Medium with ROCK inhibitor (Stem Cell Technologies,Y-27632, 10 μM) was added into each well of a 6 well plate. Secondly, the vector mix (4 μg in total, dCas9-p300: sgRNA is 2 : 1 with 2.6μg dCas9-p300 and 1.4 μg sgRNAs), 12 μl of Transit-LT1 (Mirus Bio) and 400 μl of Opti-MEM were mixed and incubated at room temperature for 30 minutes. During the incubation, iPSCs were dissociated into single cells using accutase (Gibco™, 6 mL) at 37ºC for 4 minutes. An equal volume of media (6 mL) was added, cells were centrifuged at 300g for 3min, and washed once with culture media to thoroughly remove residual accutase. iPSCs or embryonic stem cell were diluted to 500,000 cell/mL using Essential 8™ Medium with Rock inhibitor and 1 mL was added to 400μl of Transit-LT1 vector mix in each well.

HEK-293T cells were cultured in DMEM 4.5g/L glucose without L-Glutamine (Lonza) supplemented with 10% fetal bovine serum, 1% GlutaMAX (Life Technologies) and 1% Non-Essential Amino Acids Solution (Life Technologies) at 37 °C and 5% CO_2_. For transfection experiments, 5x10^5^ HEK-293T cells were plated into 6-well plates one day before transfection. In total, 4 μg of vector mix was transfected using 12 μl Transit 2020 (Mirus Bio) and 400 μl opti-MEM (Invitrogen). The vector mix (4 μg in total, dCas9-p300 : sgRNA is 2 : 1 with 2.6μg dCas9-p300 and 1.4 μg sgRNAs) were used.

Both stem cell samples and HEK-293T cell samples were harvested 48 hours post transfection and subsequently processed with RNA extraction (RNaeasy kit, Qiagen), reverse transcription (QuantiTect Reverse Transcription Kit,Qiagen) and qPCR detection (SYBR Green Real-Time PCR Master Mixes, Invitrogen) for target gene expression. The identity of the endogenous targeting sgRNAs and qPCR primers were listed in the table below. All sgRNAs were cloned into backbone (Addgene:67990) using Zhang lab protocol.^39^^,^^40^ The same group of sgRNAs were used both for dCas9-VPR and dCas9-p300 experiments.

**Table:** 

sgRNA oligo name	sgRNA oligo sequence	sgRNA name on Figure S2
Ascl1_sgRNAa_FWD	caccgCGGGAGAAAGGAACGGGAGGgt	Ascl on target
Ascl1_sgRNAa_REV	taaaacCCTCCCGTTCCTTTCTCCCGc	
NeuroD1_sgRNAd_FWD	caccgGGTCCGCGGAGTCTCTAACgt	NeuroD1
NeuroD1_sgRNAd_REV	taaaacGTTAGAGACTCCGCGGACCc	
scramble_sgRNAa_FWD	CACCGCTGAAAAAGGAAGGAGTTGAGT	Scramble sgRNA1
scramble_sgRNAa_REV	TAAAACTCAACTCCTTCCTTTTTCAGC	
scramble_sgRNAb_FWD	CACCGAAGATGAAAGGAAAGGCGTTGT	Scramble sgRNA2
scramble_sgRNAb_REV	TAAAACTCAACTCCTTCCTTTTTCAGC	

**Table:** 

oligo name	oligo sequence
human Ascl1 FWD primer	CGCGGCCAACAAGAAGATG
human Ascl1 REV primer	CGACGAGTAGGATGAGACCG
human NeuroD1 FWD primer	GGATGACGATCAAAAGCCCAA
human NeuroD1 REV primer	GCGTCTTAGAATAGCAAGGCA

#### Genotyping for reporter

To map the reporters to a genomic locus, we applied an inverse PCR method. Briefly, we first extracted genomic DNA from iPSC cells using AllPrep DNA/RNA Mini Kit (Qiagen). 5 μg of DNA were digested with either *Tat* I (Thermo) or *Msp* I at 37 ºC or 65 ºC overnight in a volume of 40 μl. We used two enzymes to achieve better coverage of the genomic sequences. Independent replicates were generated using 3 concentrations of the enzyme (60 units, 120 units and 180 units) in order to prevent potential over or under digestion. Subsequently, all DNA was purified using DNA Clean & Concentrator™-25 (Zymo Research). To encourage circularisation, 1 μg DNA was diluted to 2 ng/μl and ligated with T4 ligase (4000 unit, NEB) overnight at 16ºC and purified by DNA Clean & Concentra/tor-5 Kit (Zymo Research). Inverse PCR was carried out with primers at the beginning of the SV40 poly A signal (GTGACTGGAGTTCAGACGTGTGCTCTTCCGATCTCTGCATTCTAGTTGTGGTTTGTCC) and at the end of the piggybac 5’ end long terminal repeat (LTR) (ACACTCTTTCCCTACACGACGCTCTTCCGATCTACGCAGACTATCTTTCTAGGGTTAA). The underlined sequences are the sequence binding to the SV40 and LTR and the italic sequences are part of the i7 and i5 illumina sequencing adapter. Finally, 23 cycles of inverse PCR were carried out using KAPA HiFi HotStart ReadyMix (KAPA Bioscience) at 55 ºC with 1 min elongation time. To add the illumina P5 and P7 adapters, a second round of PCR was carried out with P5 primer: AATGATACGGCGACCACCGAGATCTACACNNNNNNNNACACTCTTTCCCTACACGACGCTCTTCCGATCT and P7 primer:CAAGCAGAAGACGGCATACGAGATNNNNNNNNGTGACTGGAGTTCAGACGTGTGCTCTTCCGATCT for 5 cycles at 67ºC annealing temperature. Here, the N represents the sample specific indices.

#### Single-cell CRISPR activation experiment

At 48 h post-transfection, three cell pools were generated: all cells containing on-target sgRNAs and dCas9-VPR, all cells containing scramble sgRNAs and dCas9-VPR and non-transfected cells. To enrich positively transfected cells, we used FACS to sort out both the cherry and BFP positive cells, which indicated successful transfection of dCas9-VPR and sgRNA vectors respectively. Finally, we pooled these three pool of cells together (no transfection: scramble sgRNA: on-target sgRNA = 5:5:90) and generated both sgRNA library and transcriptome library using Chromium Single Cell 5′ Reagent Kits V2 chemistry (10x genomics) with direct guide capture using a spiked in sgRNA specific RT oligo as previously described.^28^^,^^29^ In total, 4 lanes of 5’ end V2 kit were used. 40,000 cells were pooled together to obtain ∼400 cells for each CRISPRa perturbation. We sequenced 4 sgRNA libraries using miSEQ (Illumina) and 4 transcriptome library using NovaSEQ S4 (Illumina).

### Quantification and statistical analysis

#### Processing single cell CRISPRa experiment data

To analyse single cell CRISPRa data, we first used CellRanger software (10x Genomics) to map reads, generate UMI counts, call cells and sgRNAs. Downstream customized analyses were performed in Python, using a combination of Numpy, Scipy, Pandas, scikit-learn, and seaborn libraries. Briefly, sgRNA data were first mapped to individual cells. Here, we only included cells containing 1-5 sgRNAs since we delivered 5 sgRNAs to target each endogenous gene. We then use Scrublet to call and remove doublets.^42^ Data were filtered by no more than 10 mitochondria reads per cell and cell count was normalised to 10,000 reads per cell. Subsequent UMAP and analysis were mainly performed with scanpy and seaborn packages.

#### Mathematical modeling of gene expression

All mathematical models of zero states across chromHMM groups were performed with custom scripts in R. We assume that each cell could be in either of two latent states: “basal” and “active”. We then modeled the observed target gene UMI counts of each cell using a negative binomial distribution, with the ChromHMM and the latent states as predictors and the logarithmic total UMI counts as an offset. Since the latent states are unobserved, we obtain the maximum likelihood estimators of the regression coefficients through an expectation maximisation (EM) algorithm, which iteratively assigns soft latent state labels for each cell and fits a negative binomial model using the current latent state labels, until convergence. The final negative binomial regression coefficients and overdispersion parameters can be used to compute the mean and the variance of UMI counts for each ChromHMM class. The coefficient for activation shows the log fold increase of the mean expression between the basal and active latent states, and is an indication of the CRISPRa activation outcome. For instance, a ChromHMM class 1 cell in a basal state when sequenced with a total UMI count of 10,000 is expected to have a target gene expression of $e^{-13.49+\log\left(10000\right)}=0.014$, whereas the same cell in an active state has a mean target gene expression of $e^{-13.49+3.94+\log\left(10000\right)}=0.71$. The dispersion parameter indicates the over-dispersion of variance relative to the mean in the negative binomial distribution.

#### Expression of reporters

In order to evaluate the barcoded reporter expression levels, we carried out targeted PCR and next generation sequencing for both genomic DNA and reverse transcribed RNA samples. We first extracted both DNA and RNA from the same sample using AllPrep DNA/RNA Mini Kit (Qiagen). DNA and RNA were then quantified by Nanodrop and subsequently diluted into equal concentration. In order to remove residual DNA contamination, 1 μg of RNA were treated with TURBO^TM^ DNase (Thermo) following the standard protocol. RNA was reverse transcribed (RT) using the QuantiTect Reverse Transcription Kit (Qiagen) with a gene specific RT primer (CTGCATTCTAGTTGTGGTTTGTCC) mapping immediately downstream of the barcode. Both DNA and reverse transcribed RNA were then amplified with primers flanking the up and down stream of the barcode loci. The first step PCR was carried out at 60 ºC annealing temperature for 18 cycles with primers ACACTCTTTCCCTACACGACGCTCTTCCGATCTAGCAAAGACCCCAACGAGA and GTGACTGGAGTTCAGACGTGTGCTCTTCCGATCTTGGTTTGTCCAAACTCATCAATGTATC. Underlined sequences indicate the reporter binding region, while italic sequences are part of the Illumina sequencing adapter. In total, 1-2 μg of gDNA or cDNA were amplified using KAPA HiFi HotStart ReadyMix (KAPA Bioscience) with 100μl or 200μl reaction volume. To minimise amplification bias, each PCR was equally split into 4 reactions. To add llumina P5 and P7 adapters, a second round PCR was performed at 67ºC annealing temperature for 24 cycles with Primer1: AATGATACGGCGACCACCGAGATCTACACNNNNNNNNACACTCTTTCCCTACACGACGCTCTTCCGATCT and primer 2: CAAGCAGAAGACGGCATACGAGATNNNNNNNNGTGACTGGAGTTCAGACGTGTGCTCTTCCGATCT. Ns represent the sample specific barcodes. Finally, all the gDNA and cDNA target amplicons were sequenced using Illumina miSEQ platform.

#### Processing the RNA-Seq and ChIP-Seq data

We cloned 480 sgRNAs in an arrayed format following previously described protocols^40^ and extracted plasmid using QIAprep Spin Miniprep Kit (Qiagen). Subsequently, we pooled all 5 sgRNAs targeting the same gene equally and further purified using DNA Clean & Concentrator-5 (Zymo Research). The 5 sgRNA vectors were pooled, together with purified dCas9-VPR construct and transfected into A1ATD iPSC cells in 96 well plate format. As a negative control, a plate of dCas9-VPR and a mixture of 2 scramble sgRNA vectors were used. Briefly, for each 96 well, 250ng of DNA (dCas9-VPR : pool of sgRNAs for one gene 0.65:0.35) were mixed and incubated together for 20-–30 minutes in 0.75μl Transit LT1 and 25 μl serum free media (Opti-MEM Reduced Serum Medium, Thermo Fisher scientific). During the incubation, one 10cm dish of iPSCs were dissociated into single cells using 6 mL Accutase (Gibco) at 37ºC for 4 minutes. Upon single cell digestion, an equal volume of media (6 mL) was added, cells were centrifuged at 300g for 3min, and washed once with culture media to thoroughly remove residual Accutase. iPSCs were diluted into 500,000 cell/mL using Essential 8 Medium with Rock inhibitor (Stem Cell Technologies,Y-27632, Final concentration 10nM) and 12.5μl cell (35K cells per well) was added into 25μl Transit-LT1 mix contained well. Media was changed at 24 h post transfection into normal E8 media without Rock inhibitor.

All RNAseq data was quantified using Salmon with human GRCh38 cDNAs. Transcript quantification and downstream analysis was carried out using R package readr, tximportData and DESeq2 and we removed transcripts with raw counts less than 10. PCA plots were generated with R package pcaExplorer. Differential expression tests were carried out between time point 0 hrs and all other times with minimum |log_2_ (fold change)| > 2 and adjusted p value < 0.001. Gene set enrichment analysis for each time points were carried out using g:Profiler.^43^

All ChIP data was first mapped to the hg38 genome using HISAT2. Peaks were then called using macs2 with extsize 200bp, SPMR normalization and qvalue 0.01. For H3K36me3 and H3K27me3, the “broad” flag was used. Noise-subtracted and normalized pileup signals were generated by macs2 bdgcmp. After converting bdg to bigwig, Deeptools was then used to compute the matrix of peak signal surrounding the transcription start site (TSS) and transcription end site (TES). A customized python script was then used to plot the mean and 95% confidence interval of 3 technical replicates.

#### Processing reporter data

We first analyzed genotyping data to map barcode insertion site. Secondly, we used targeted gDNA and cDNA sequencing data to generate the normalized barcode expression levels. Thirdly, all data was merged and only reporters with location and expression data were retained for downstream analysis.

##### Genotyping analysis

All paired end genotyping reads were first merged together. A customized python script was used to filter the reads with a specific structure containing piggybac sequences, barcode upstream sequences and barcode downstream sequences. Then, all the reads with an incorrect barcode length were removed and barcode sequences were moved to the header of the reads. Piggybac related sequences were removed and the rest of the read was then mapped to the hg38 genome using HISAT2. The number of read counts of each mapped barcode were generated by a customized python script. When one barcode was mapped to multiple locations, we first examined whether the mapping distance is within 10bp. If so, we merged the counts together and used the midpoint as their mapping location. After merging, if the reporters remain mapped to multiple locations, we defined them as ambiguous and placed a flag in the ambiguity column. Two enzymes (Tat1 and MspI) were used, and we included barcodes that can be mapped at least by one condition of the enzymatic digestion. Only unambiguous barcodes were then used in the following downstream analysis.

##### Expression analysis

To analyse the expression data, all paired end reads were merged together using FLASH.^44^ Barcodes were then extracted using customized python script by finding the reads with correct structure including piggybac sequences, barcode upstream sequences and barcode downstream sequences to generate aclean file containing barcode identity (.bc). We then paired the barcodes in gDNA with those in cDNA and calculated the barcode expression score using a customized python script. We filtered out all barcodes with the total gDNA and cDNA count less than 100 and expression score was calculated as sum all cDNA count / sum of gDNA count within one biological replicate. In order to segment barcode expression into the 4 groups shown in Figure 2D we used the basal barcode expression and the day 5 to day 0 barcode expression fold change. For Groups 1, 2, and 3, both iPSC and iNeuron samples contain detectable barcode expression. Group 1 consists of barcodes with a fold change 0 to 0.5 (Day5 to Day1) (turned off), Group 2 contains barcodes with a fold change 0.5 to 2 (always on) and Group 3 has those barcodes with a fold change bigger than 2 (turned on). Group 4 (constitutively off) contains all other barcodes that in iPSC and/or iNeuron contain undetectable levels of barcode expression. Note that all group 4 barcodes are detectable at the gDNA level, but not in cDNA. The z-score describes the fold change of observed values to the mean of all values in one group.

##### Integrating reporter location and expression data and other downstream analysis

In total, 3986 barcodes contain either location or expression information. Next, we filtered to leave 2979 barcodes which were unambiguously mapped to a unique genomic location. We then normalized the iPSC and iNeuron expression with a batch normalization factor (median of ratios method). Finally, we filtered to leave the barcodes with at least two independent non-zero observations. In this study, we used these 2923 mapped barcodes except on Figures 3G–3N. When building a basal expression versus activation model, we only evaluated the expressed and well-represent barcodes, hence, we filtered out the less represented barcodes (total gDNA read count <10) and non-expressed barcodes (total cDNA read count without CRISPR activation is 0). In Figures 3G–3N, a total of 1151 barcodes in iPSCs and 1050 barcodes in iNeurons are shown.

##### Putative safe harbour analysis

We first excluded barcodes with less than 20 read counts in both gDNA and cDNA datasets then removed any barcodes for which the inverse PCR mapping has less than 30 read counts. Then, we only include genomic insertion sites that map unambiguously to a single site and selected integrations within intergenic and intronic regions. Finally, we validated each location manually and assigned a genomic region annotation. Results are shown in Table S1.

### Additional resources

ChromHMM data is integrated into our CRISPR design tool website to enable selection of optimal guides for CRISPRa experiments: https://wge.stemcell.sanger.ac.uk/

## Data Availability

• Single-cell RNA-seq data have been deposited at EGA and are publicly available as of the date of publication. Accession numbers are listed in the key resources table. • All original code has been deposited (Zenodo: 10.5281/zenodo.7650425) and is publicly available as of the date of publication. DOIs are listed in the key resources table. • Any additional information required to reanalyze the data reported in this paper is available from the lead contact upon request.

## Supplemental information

Document S1. Figures S1–S5 and Data S1–S4

Table S1. Summary of barcode expression and location, related to Figures 1, 2, and 3 Read counts of individual biological repeats for each reporter barcode in cDNA or gDNA and cDNA to gDNA ratios under different conditions. This includes days 0, 2, and 5 of differentiation (D0, D2, and D5), targeting (SCP) or non-targeting (SCR) guide sets in iPSC or iNeuron cell types. Mapping chromosome (loc_chr) and location (loc_location) is also shown along with the number of reads that defined this location (sum_sum) and those mapping from each mapping experiment (Tat1 and Msp1 at different concentrations). Segment numbers indicate cluster group in Figure 2B.

Table S2. sgRNA sequences for single cell CRISPRa experiment, related to Figures 4, 5, and 6 Guide ID number, name, 20 nt protospacer sequence (excluding PAM), and targeted gene for each guide used in the CRISPRa experiments is indicated.

Document S2. Article plus supplemental information
