# Supplementary material for: Massively parallel characterization of CRISPR activator efficacy in human induced pluripotent stem cells and neurons
Source: Mol Cell. 2023 Apr 6;83(7):1125–1139.e8. doi: 10.1016/j.molcel.2023.02.011 (PMC10114495; doi:10.1016/j.molcel.2023.02.011)

Molecular Cell, Volume *83*

Supplemental information

Massively parallel characterization of CRISPR activator efficacy in human induced pluripotent stem cells and neurons

Qianxin Wu, Junjing Wu, Kaiser Karim, Xi Chen, Tengyao Wang, Sho Iwama, Stefania Carobbio, Peter Keen, Antonio Vidal-Puig, Mark R. Kotter, and Andrew Bassett

Supplemental Information

Supplemental Figure Legends

Figure S1: Transcriptional and chromatin changes during differentiation of iPSCs to iNeurons, related to figure 1

a) Schematic diagram for iPSC and iNeuron culture and transfection **b)** Principal component analysis of transcriptomic data during iNeuron conversion (n=3 for each time point) showed synchronous and extensive transcriptional changes during differentiation **c)** ChIP-Seq peak number analysis of six post-translational modifications of histones that have been used previously to segment the genome into 10-15 chromatin states (ChromHMM[21]) in iPSCs and iNeurons. **d)** ChIP-Seq peak coverage analysis **e)** Top panel: ChIP-seq data from iPSCs and iNeurons (data shows mean and 95% confidence interval of 3 technical replicates). Bottom panel: ChIP-seq analysis showing gene body and 5 kb up and downstream using iPSC and iNeuron data. Genes were scaled within the region of transcription start site (TSS) and transcription end site (TES) (red and green dotted lines). **f)** Left panel: RNA-seq heatmap of significantly up (top, n=1660) and down (bottom, n=1803) regulated genes (Benjamini-Hochberg adjusted, FDR < 0.001) during conversion. Heatmaps show regularized log transformed RNA seq read counts across 4 time points (from left to right: 0 , 24, 48 and 96 hours post-induction). The line graphs show four marker genes (coloured lines). Markers of neuronal cell fate (NeuroD1, PAX6, SOX1, SYP) were upregulated and markers of pluripotency (NANOG, MYC, ZFP42, LIN28A) were downregulated during this time course**.** The grey dotted lines show the mean of all genes within the up and down regulated groups and standard deviation of 3 biological replicates. Right panel: Metagene analysis of ChIP-seq data of significantly changed groups of genes. The solid lines show the mean peak intensity at iPSC (blue) and iNeuron (red) stages of 3 replicates and 95% confidence intervals. **g)** Gene set enrichment analysis of significantly up and down regulated genes at each time point compared to day 0 (Benjamini-Hochberg adjusted, FDR < 0.001). The bar plot shows the number of significantly up (left) and down (down) regulated genes at each time point. The dot plot shows the top 20 ranked pathways (dot size and colour indicates p-values). As expected, pathways related to nervous system development (e.g. synaptic transmission, nervous system development) were present in the "turned on"set and those related to stem cell function (e.g. developmental process, cell differentiation) in the "turned off" set.

Figure S2: dCas9-p300 fails to activate gene expression at stem cell stage, related to figure 2

a) Box whisker plots showing the normalised endogenous genes neighbouring reporter insertion sites (4 time points in total, 3 biological replicates for each) averaged across genes located within the specified windows up and downstream of reporter insertions. Plots are grouped by whether the reporter is on the same strand or different strand from the endogenous gene. **b)** Expression level of reporter integrations shown as log (cDNA/gDNA) in HEK293T cells when transfected with scrambled (off target, green) sgRNAs or those targeting the SCP promoter (on target, red) co-delivered with dCas9-VPR (upper) or dCas9-p300 (lower). The three graphs are split according to basal expression level from low (1) to high (3). The dot and bar shows the median and 95% confidence interval of the data. **c, d)** The summarized overall expression level across all conditions shown as log (cDNA/gDNA) ratio for iPSCs at low (b) or

high (c) concentrations of plasmid. The dot and bar shows the median and 95% confidence interval of the data. **e)** Heatmap shows reporter expression with five experiments, the dCas9-VPR co-transfected with on target sgRNA, the dCas9-VPR co-transfected with scramble control sgRNA, no transfection, dCas9-p300 co-transfected with on target sgRNA and dCas9-p300 co-transfected with scramble control sgRNA. For each group, three biological replicates were included. **f,g)** The RT-qPCR test with endogenous gene targets Ascl1 and NeuroD1.

Figure S3: Basal expression and chromatin context influence reporter activation levels, related to figure 3

a) Barcoded reporters were segmented into equal sized bins according to basal expression level. In each bin, reporters were ranked by their activation levels and assigned a group number. We indicate 4 groups on the figure for simplicity, but 5-6 groups were used for the real analysis. All reporters belonging to the same group were pooled together and chromatin modification levels were assessed across the groups. **b, c)** The exponential decay model linking basal expression to fold activation with CRISPRa for iPSC and iNeuron.

Figure S4: Examples of barcode reporter activation in all ChromHMM defined chromatin states, related to figure 3

The top panels show the normalized ChIP-Seq data 100 kb up and downstream of the reporter insertion in iPSC **a)** and iNeuron **b)**. The exact insertion sites are labelled with dotted vertical lines. The bottom panel shows the reporter expression under three conditions: no transfection, scramble sgRNA transfection and on-target sgRNA transfection. Welch's t-tests were used to calculate p values between scramble sgRNA group and on-target sgRNA group.

Figure S5: Single cell based CRISPRa experiment, related to figure 4, 6

a) Overview of computational workflow for selecting genes and guides for the single cell CRISPRa experiment. b) The percentage of cells mapped to each CRISPRa perturbation. c) Examples of each of the four outcomes observed upon CRISPR activation. d) CellNet classification heatmaps showing the performance of the tissue type classifier for each CRISPRa perturbation. Perturbations with an identifiable effect are indicated by red arrows.

Data S1: UMAP projection for CRISPRa on-target cells and scramble control cells coloured by guide identity, related to figure 6. Cells containing CRISPRa guides for a gene are indicated in red, and controls in grey

Data S2: UMAP projection for CRISPRa on-target cells and scramble control cells coloured by target gene expression level, related to figure 6

Expression of the indicated gene for each cell is shown in red.

Data S3: CRISPRa outcome with 1,2,3,4,5 sgRNAs per cell, related to figure 4

Violin plot showing gene expression levels with cells containing 1,2,3,4,5 sgRNAs. For on-target sgRNAs, 5 sgRNAs were used. For controls, two scramble sgRNAs were used.

Data S4: CRISPR activation analysis for different sgRNA combinations, related to figure 4

CRISPR activation using all cells containing on-target sgRNAs or control scramble sgRNAs (left panel); CRISPR activation for cells containing a total number of 1,2,3,4,5 sgRNAs and scramble control sgRNA (middle panel); CRISPR activation contain each of the unique sgRNA

combinations labelled by the numbers and separated by the lines (right panel). The number 481 and 482 stands for scramble control sgRNAs 1 and 2. All other sgRNA numbers and their corresponding identity can be found in table S2.

Supplementary Figure 1

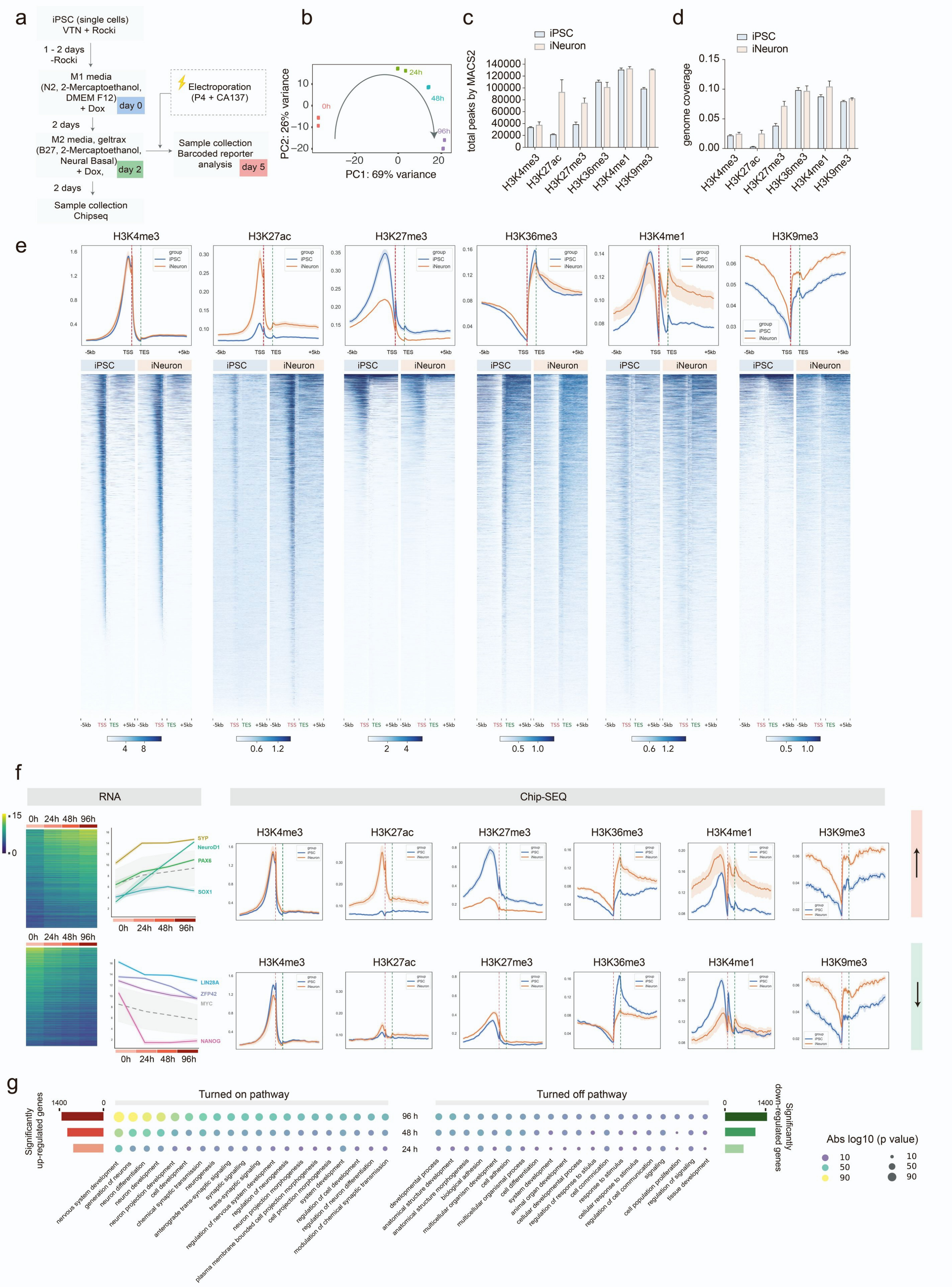

Supplementary Figure 2

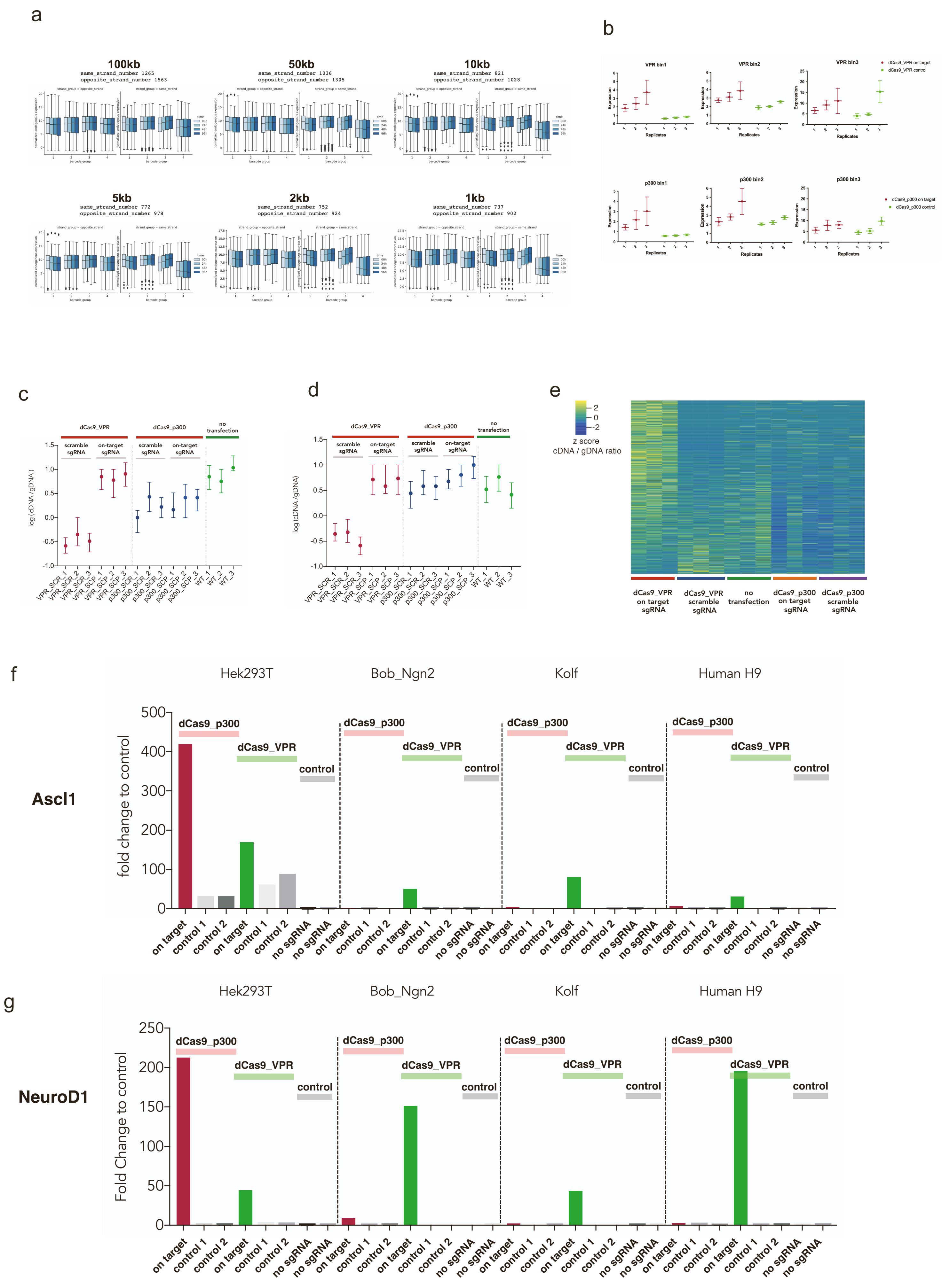

Supplementary Figure 3

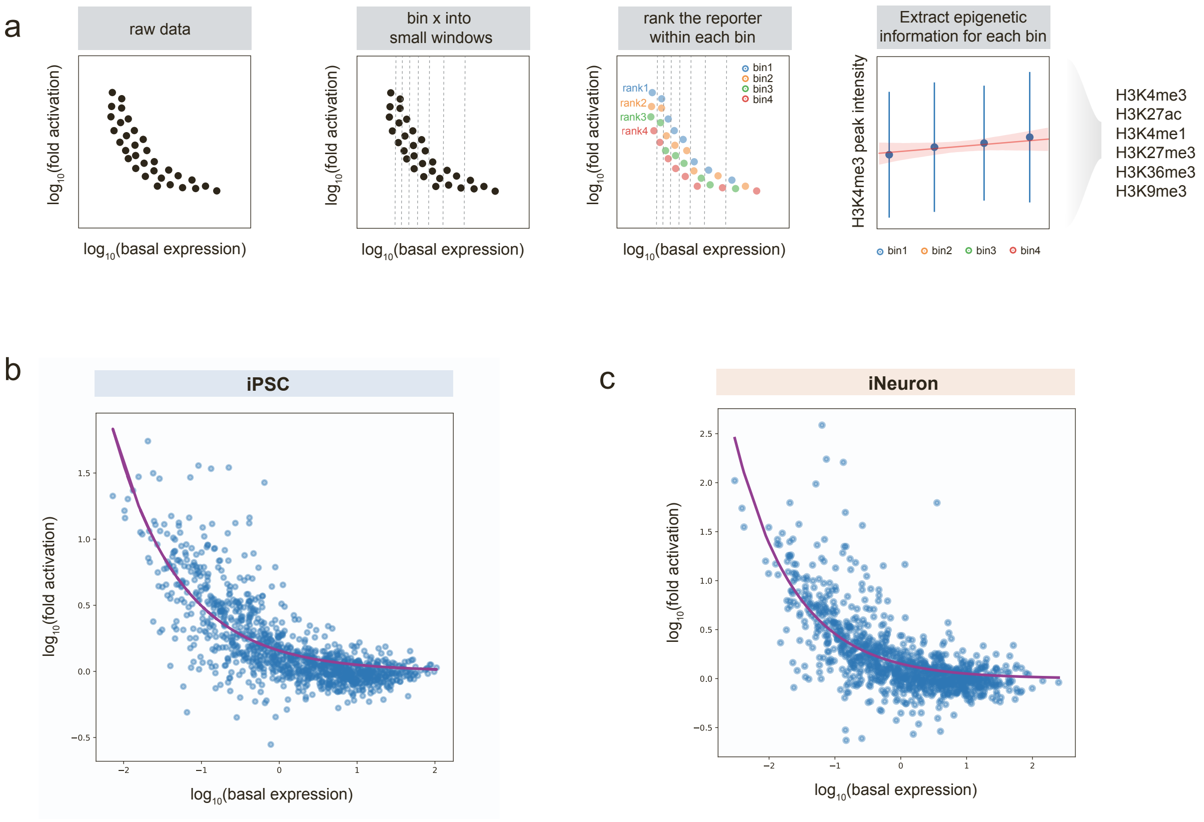

$$y = (0.157)\ e^{(-1.148) * x}$$

y: $\log_{10}$(fold activation)

x: $\log_{10}$(basal expression)

$$y = (0.152)\ e^{(-1.102) * x}$$

y: $\log_{10}$(fold activation)

x: $\log_{10}$(basal expression)

Supplementary Figure 4

a

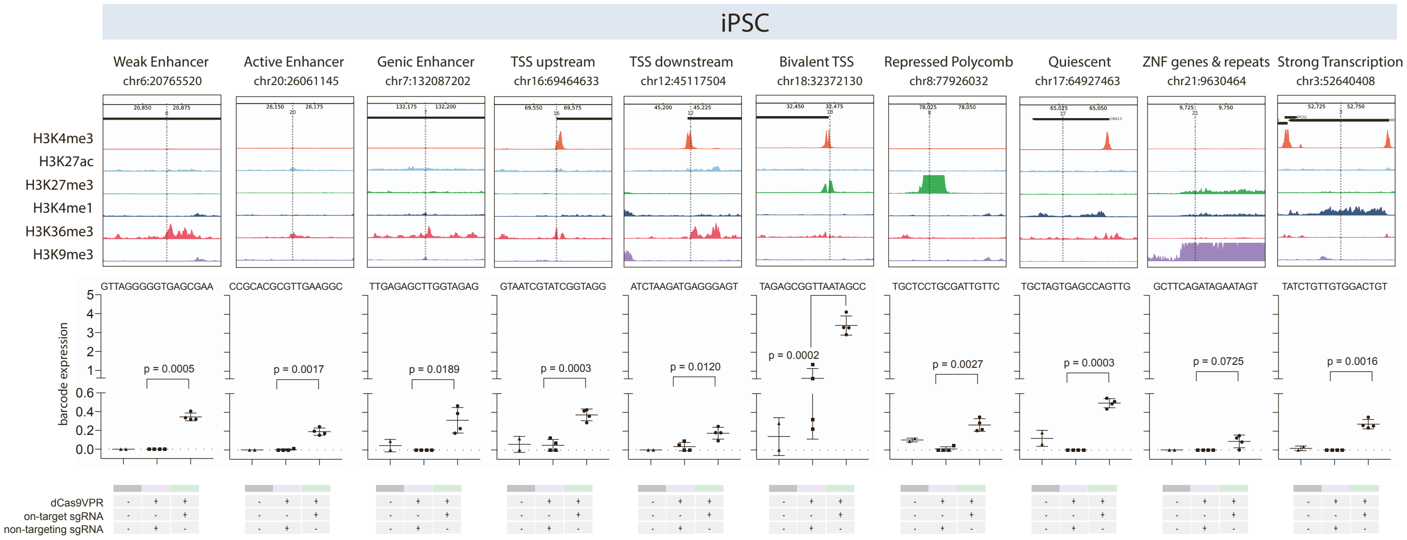

b

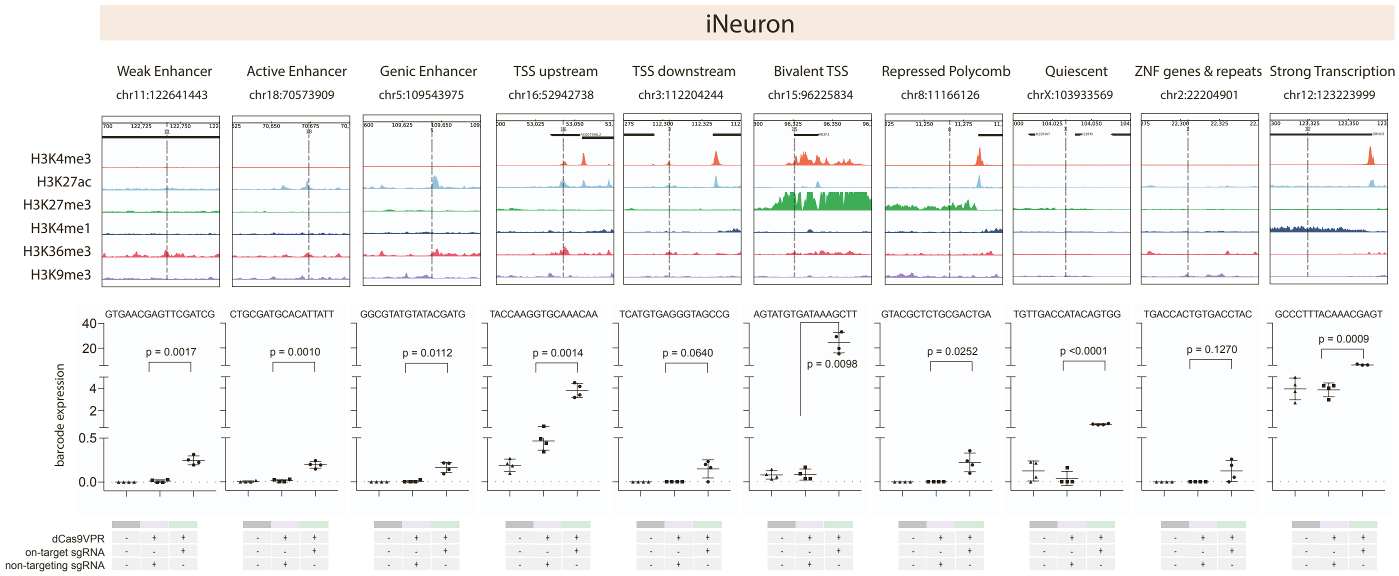

Supplementary Figure 5

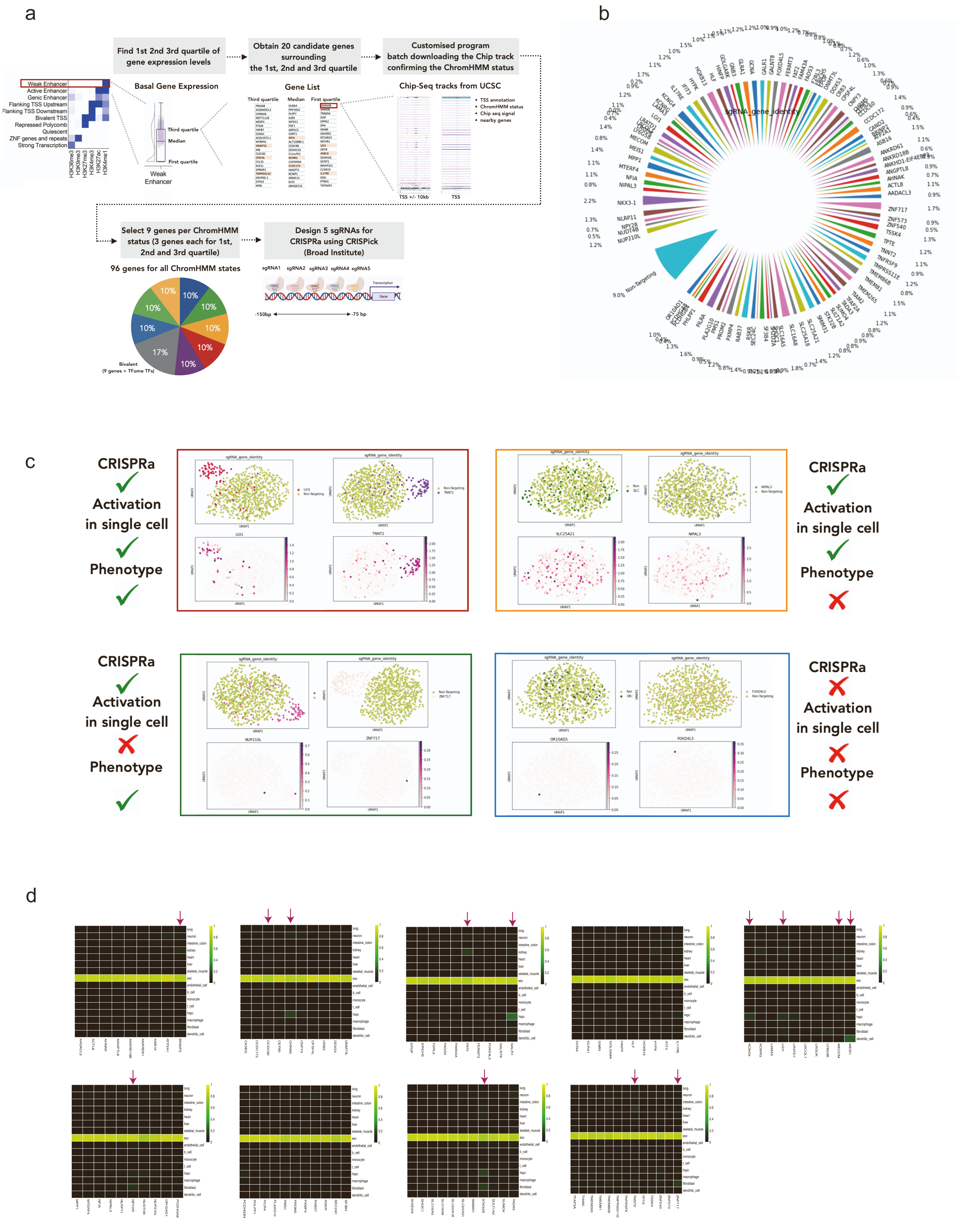

Data S1

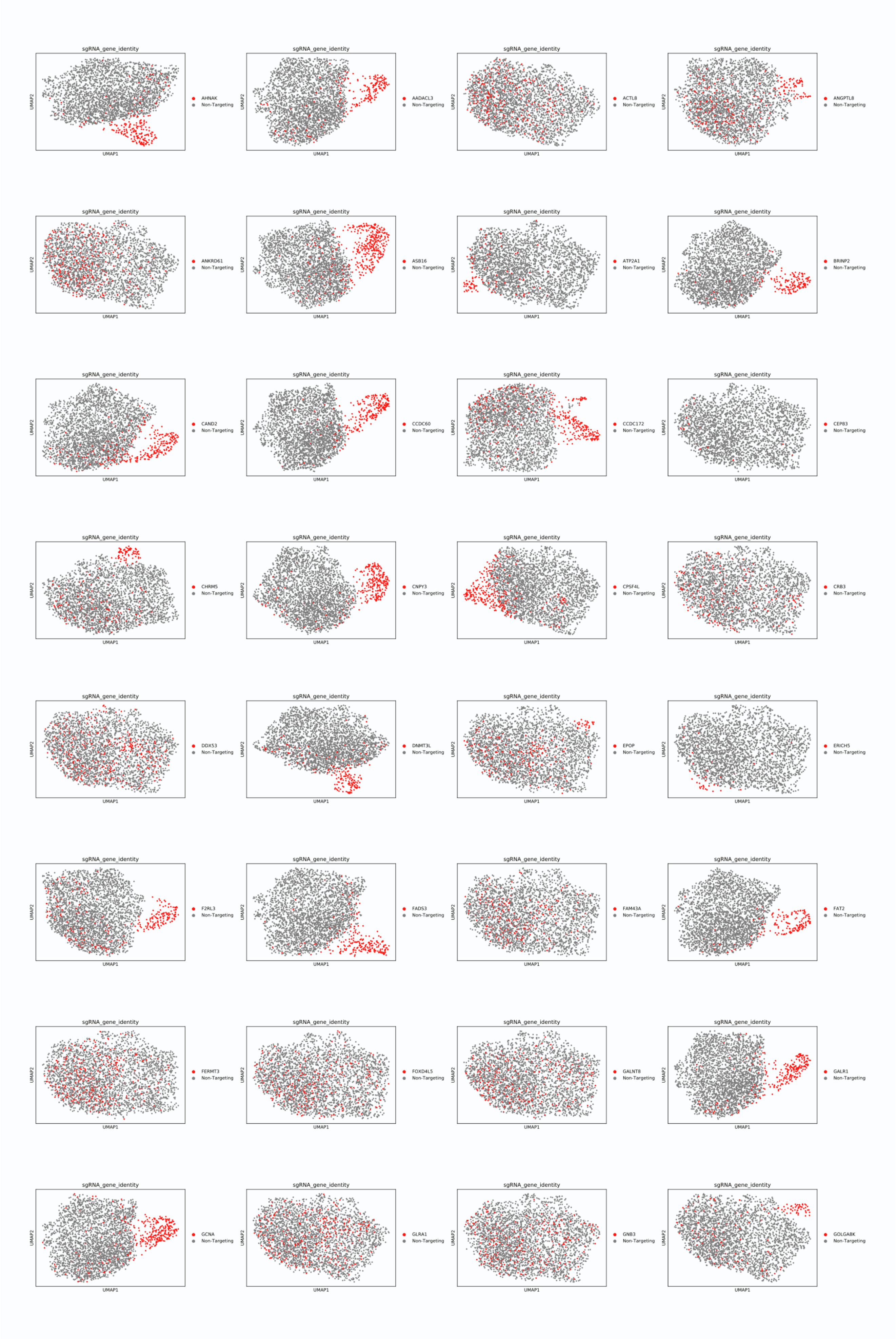
sgRNA_gene_identity
UMAP1
UMAP2
Non-Targeting
AHNAK
AADACL3
ACTL8
ANGPTL8
ANKRD61
ASB16
ATP2A1
BRINP2
CAND2
CCDC60
CCDC172
CEP83
CHRM5
CNPY3
CPSF4L
CRB3
DDX53
DNMT3L
EPOP
ERICH5
F2RL3
FADS3
FAM43A
FAT2
FERMT3
FOXD4L5
GALNT8
GALR1
GCNA
GLRA1
GNB3
GOLGA8K

Data S1

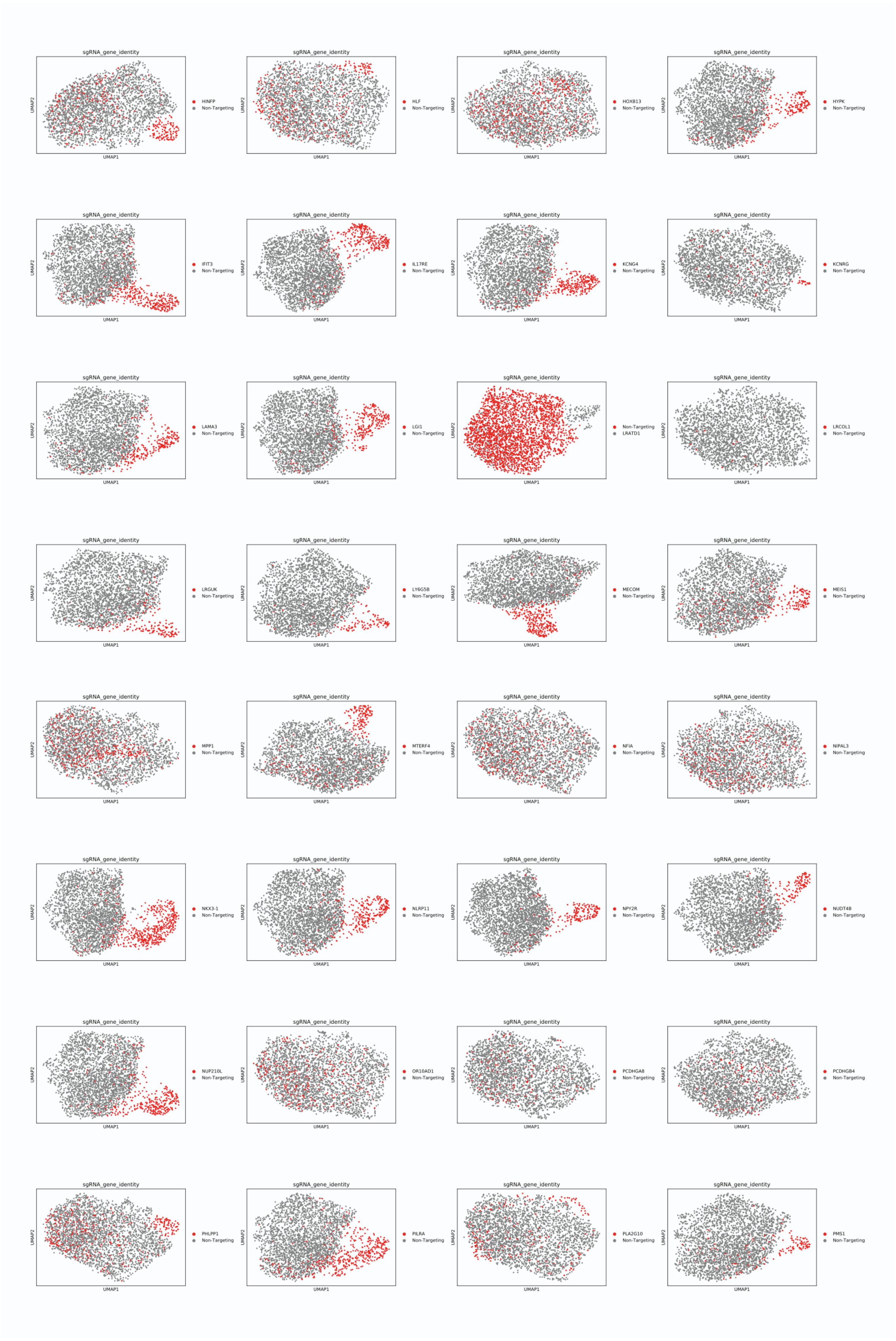
sgRNA_gene_identity
UMAP1
UMAP2
HINFP
HLF
HOXB13
HYPK
IFIT3
IL17RE
KCNG4
KCNRG
LAMA3
LGI1
LRATD1
LRCOL1
LRGUK
LY6G5B
MECOM
MEIS1
MPP1
MTERF4
NFIA
NIPAL3
NKX3-1
NLRP11
NPY2R
NUDT4B
NUP210L
OR10AD1
PCDHGA8
PCDHGB4
PHLPP1
PILRA
PLA2G10
PMS1
Non-Targeting

Data S1

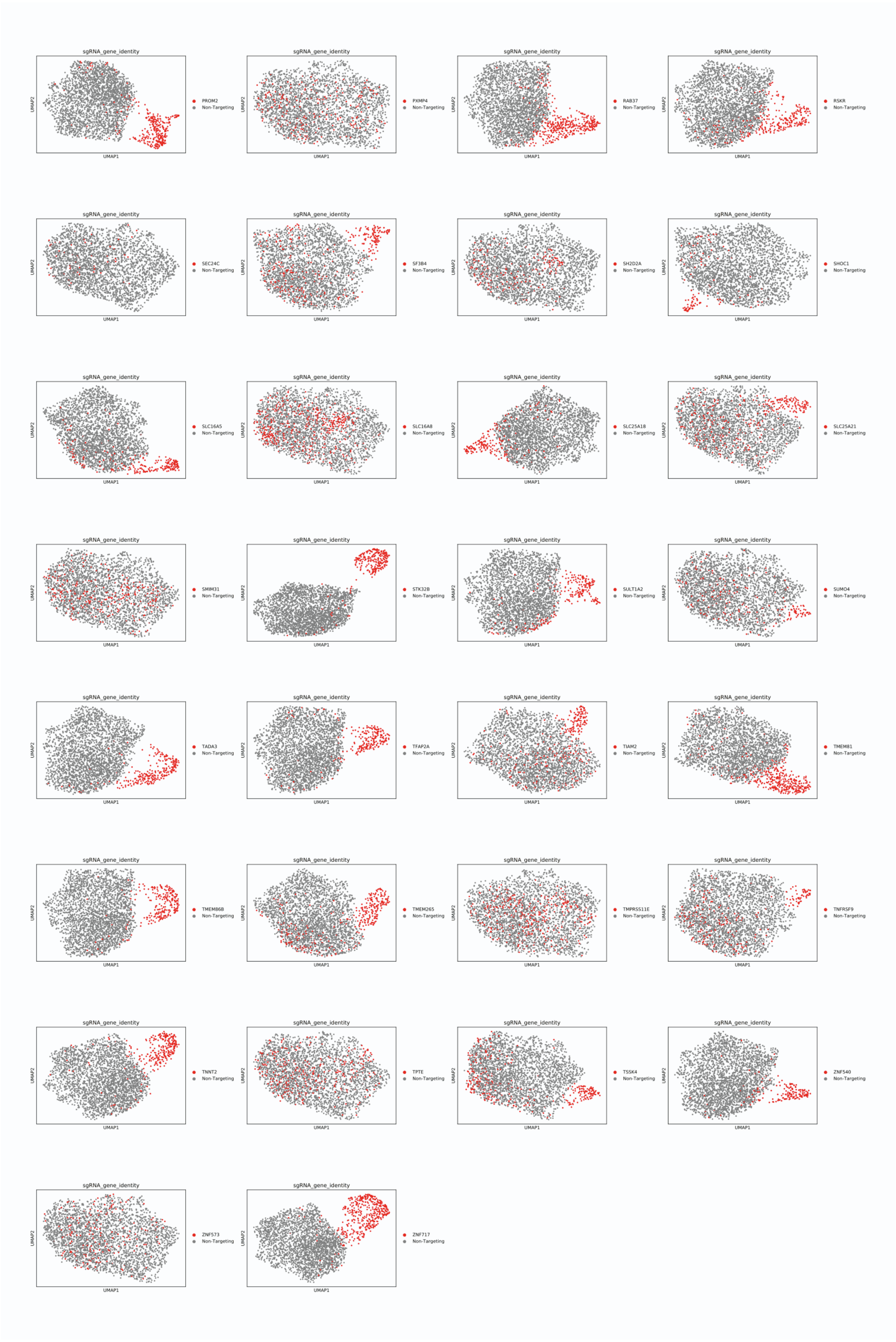
sgRNA_gene_identity
UMAP1
UMAP2
PROM2
Non-Targeting
PXMP4
RAB37
RSKR
SEC24C
SF3B4
SH2D2A
SHOC1
SLC16A5
SLC16A8
SLC25A18
SLC25A21
SMIM31
STK32B
SULT1A2
SUMO4
TADA3
TFAP2A
TIAM2
TMEM81
TMEM86B
TMEM265
TMPRSS11E
TNFRSF9
TNNT2
TPTE
TSSK4
ZNF540
ZNF573
ZNF717

Data S2

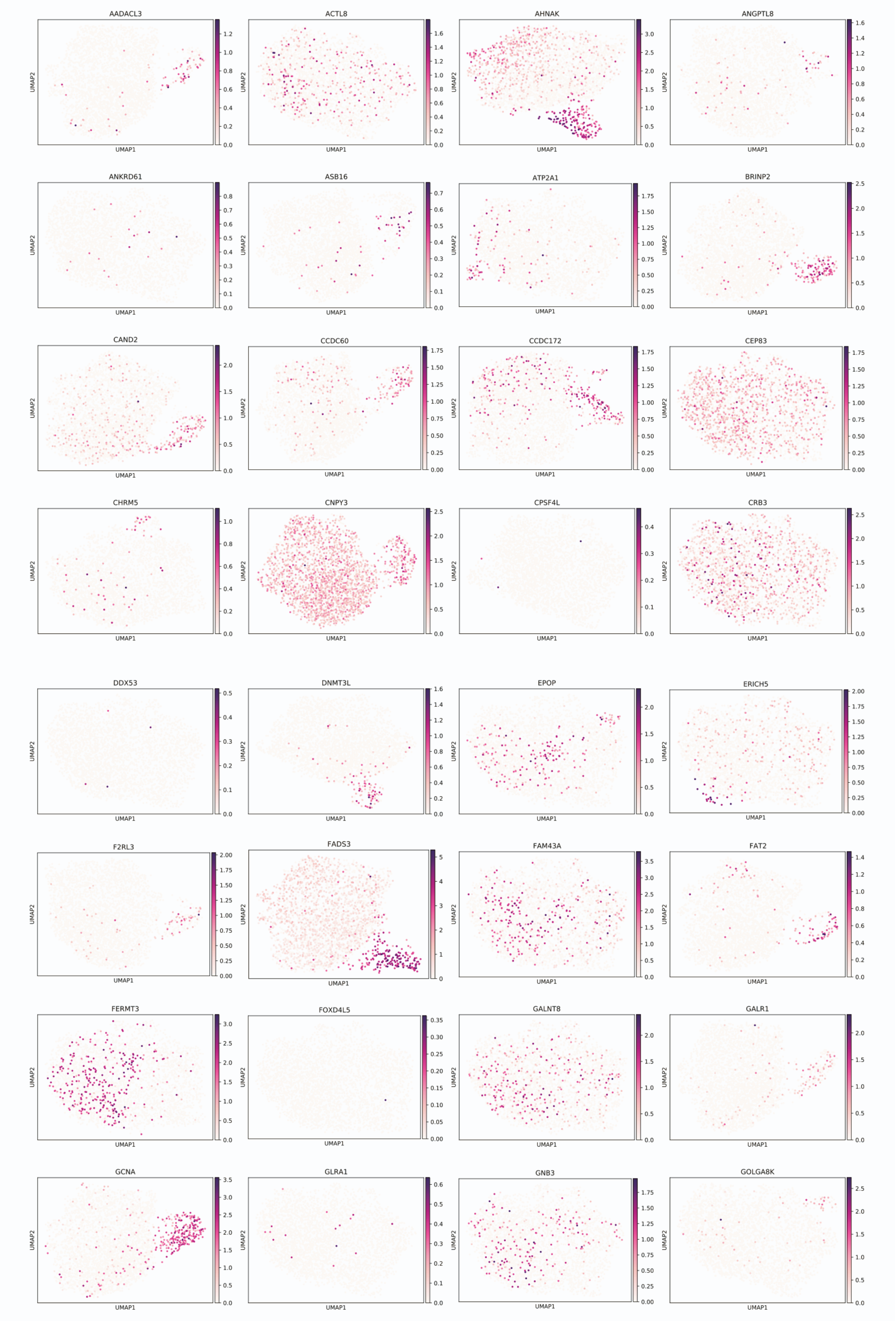

AADACL3
ACTL8
AHNAK
ANGPTL8
ANKRD61
ASB16
ATP2A1
BRINP2
CAND2
CCDC60
CCDC172
CEP83
CHRM5
CNPY3
CPSF4L
CRB3
DDX53
DNMT3L
EPOP
ERICH5
F2RL3
FADS3
FAM43A
FAT2
FERMT3
FOXD4L5
GALNT8
GALR1
GCNA
GLRA1
GNB3
GOLGA8K
UMAP1
UMAP2

Data S2

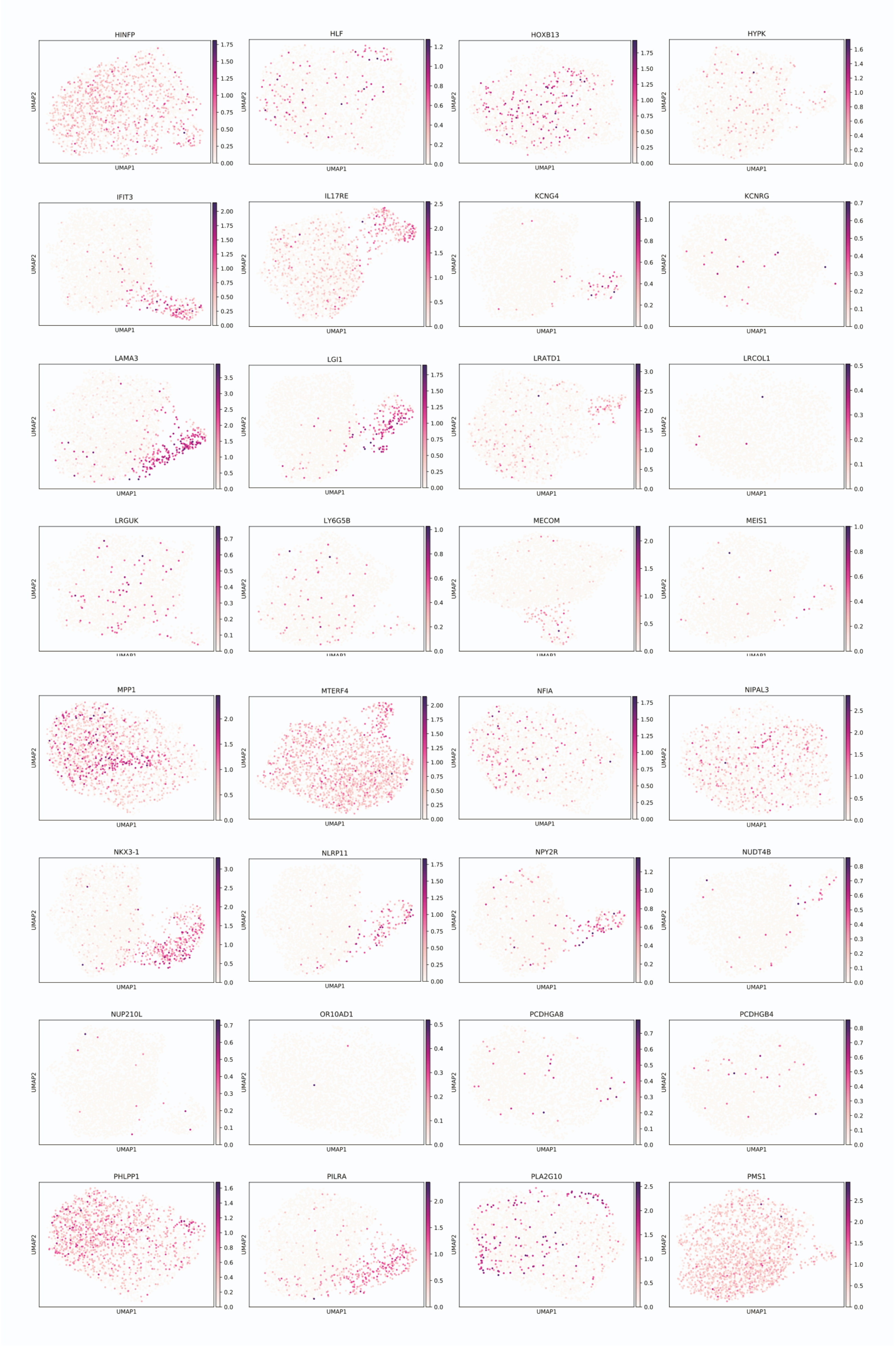
HINFP
HLF
HOXB13
HYPK
IFIT3
IL17RE
KCNG4
KCNRG
LAMA3
LGI1
LRATD1
LRCOL1
LRGUK
LY6G5B
MECOM
MEIS1
MPP1
MTERF4
NFIA
NIPAL3
NKX3-1
NLRP11
NPY2R
NUDT4B
NUP210L
OR10AD1
PCDHGA8
PCDHGB4
PHLPP1
PILRA
PLA2G10
PMS1
UMAP1
UMAP2

Data S2

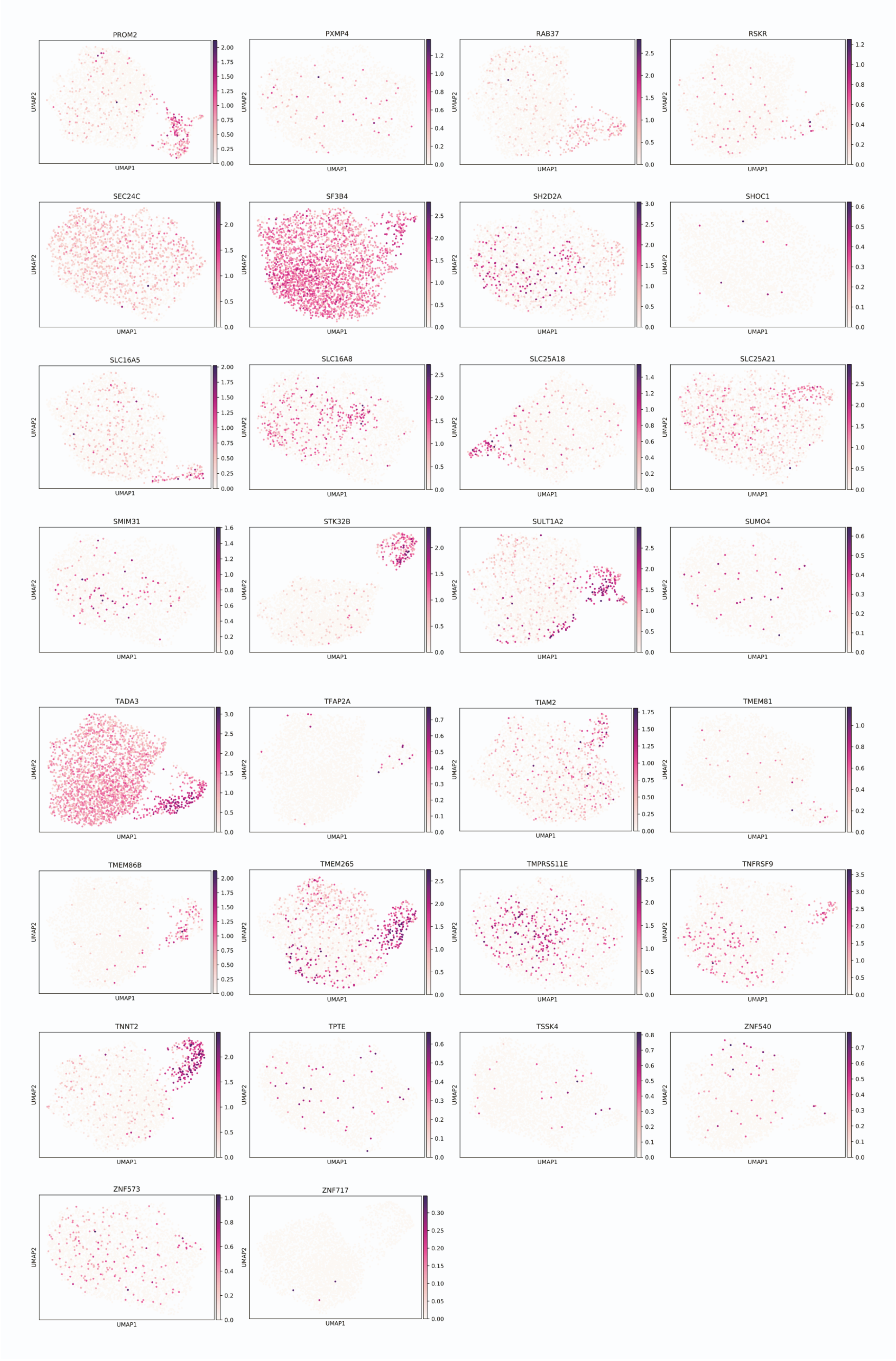
PROM2
PXMP4
RAB37
RSKR
SEC24C
SF3B4
SH2D2A
SHOC1
SLC16A5
SLC16A8
SLC25A18
SLC25A21
SMIM31
STK32B
SULT1A2
SUMO4
TADA3
TFAP2A
TIAM2
TMEM81
TMEM86B
TMEM265
TMPRSS11E
TNFRSF9
TNNT2
TPTE
TSSK4
ZNF540
ZNF573
ZNF717
UMAP1
UMAP2

Data S3

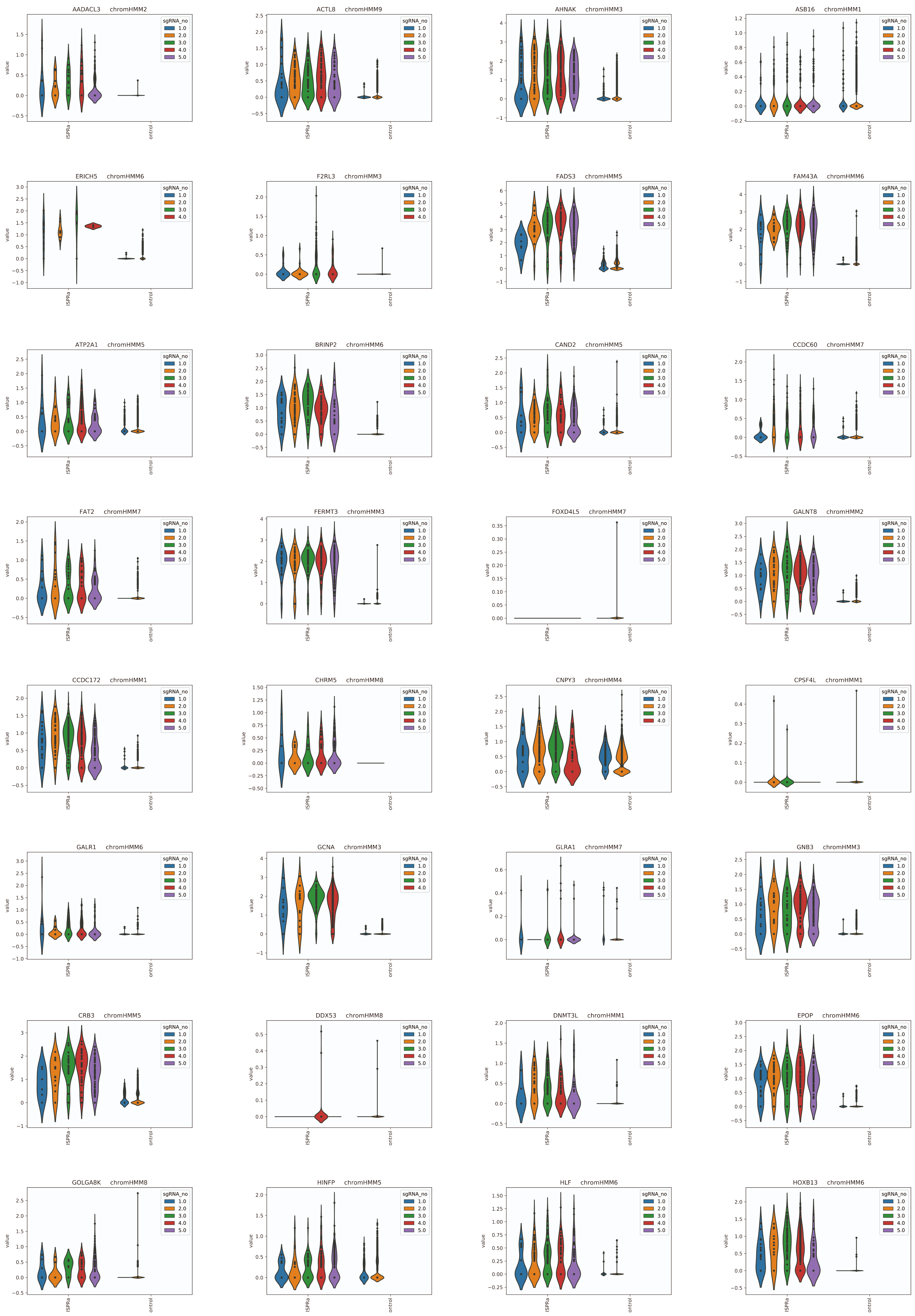
AADACL3 chromHMM2
ACTL8 chromHMM9
AHNAK chromHMM3
ASB16 chromHMM1
ERICH5 chromHMM6
F2RL3 chromHMM3
FADS3 chromHMM5
FAM43A chromHMM6
ATP2A1 chromHMM5
BRINP2 chromHMM6
CAND2 chromHMM5
CCDC60 chromHMM7
FAT2 chromHMM7
FERMT3 chromHMM3
FOXD4L5 chromHMM7
GALNT8 chromHMM2
CCDC172 chromHMM1
CHRM5 chromHMM8
CNPY3 chromHMM4
CPSF4L chromHMM1
GALR1 chromHMM6
GCNA chromHMM3
GLRA1 chromHMM7
GNB3 chromHMM3
CRB3 chromHMM5
DDX53 chromHMM8
DNMT3L chromHMM1
EPOP chromHMM6
GOLGA8K chromHMM8
HINFP chromHMM5
HLF chromHMM6
HOXB13 chromHMM6
sgRNA_no
1.0
2.0
3.0
4.0
5.0
value
ISPRa
ontrol

Data S3

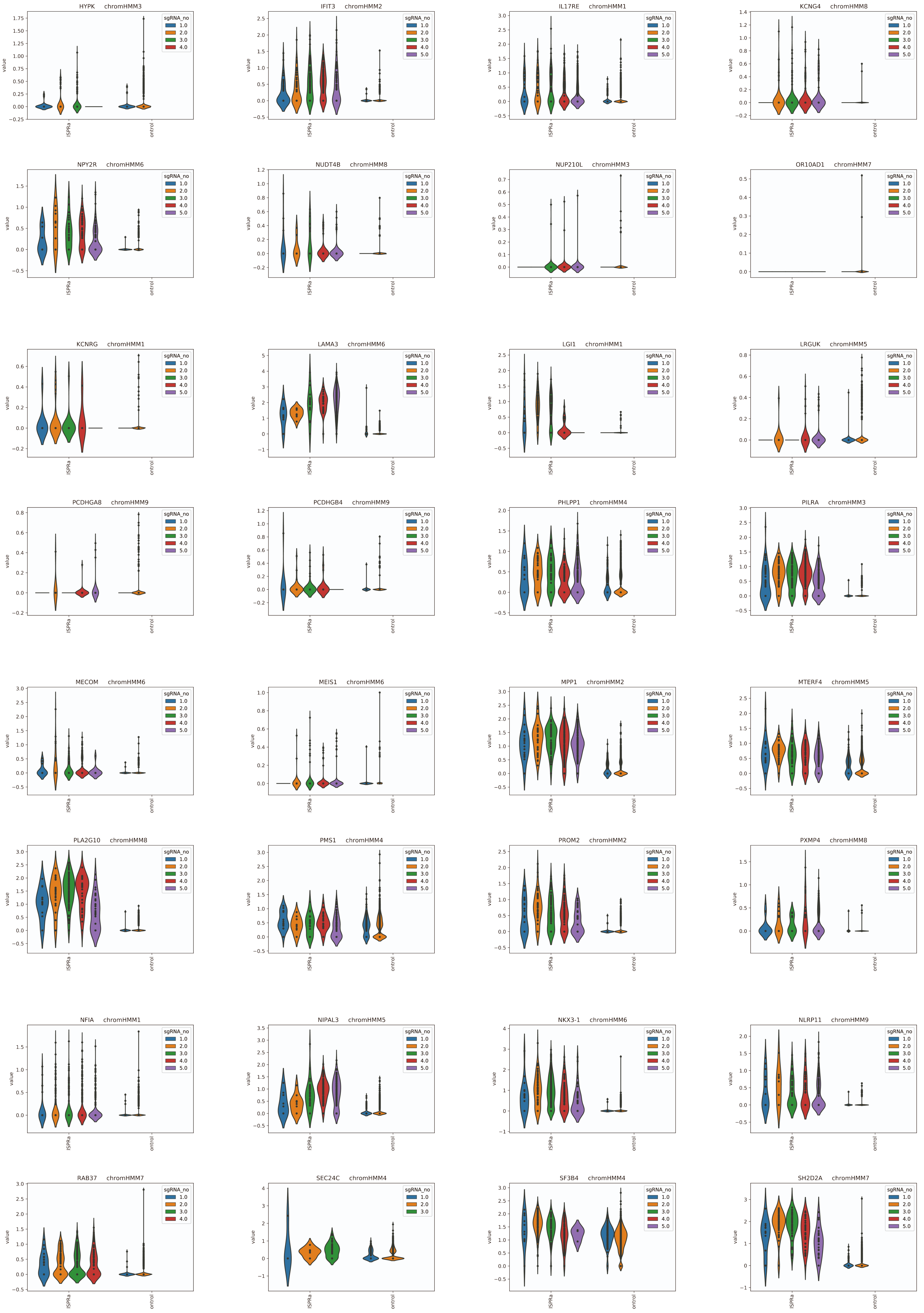
HYPK chromHMM3
IFIT3 chromHMM2
IL17RE chromHMM1
KCNG4 chromHMM8
NPY2R chromHMM6
NUDT4B chromHMM8
NUP210L chromHMM3
OR10AD1 chromHMM7
KCNRG chromHMM1
LAMA3 chromHMM6
LGI1 chromHMM1
LRGUK chromHMM5
PCDHGA8 chromHMM9
PCDHGB4 chromHMM9
PHLPP1 chromHMM4
PILRA chromHMM3
MECOM chromHMM6
MEIS1 chromHMM6
MPP1 chromHMM2
MTERF4 chromHMM5
PLA2G10 chromHMM8
PMS1 chromHMM4
PROM2 chromHMM2
PXMP4 chromHMM8
NFIA chromHMM1
NIPAL3 chromHMM5
NKX3-1 chromHMM6
NLRP11 chromHMM9
RAB37 chromHMM7
SEC24C chromHMM4
SF3B4 chromHMM4
SH2D2A chromHMM7
sgRNA_no
value
ISPRa
ontrol

Data S3

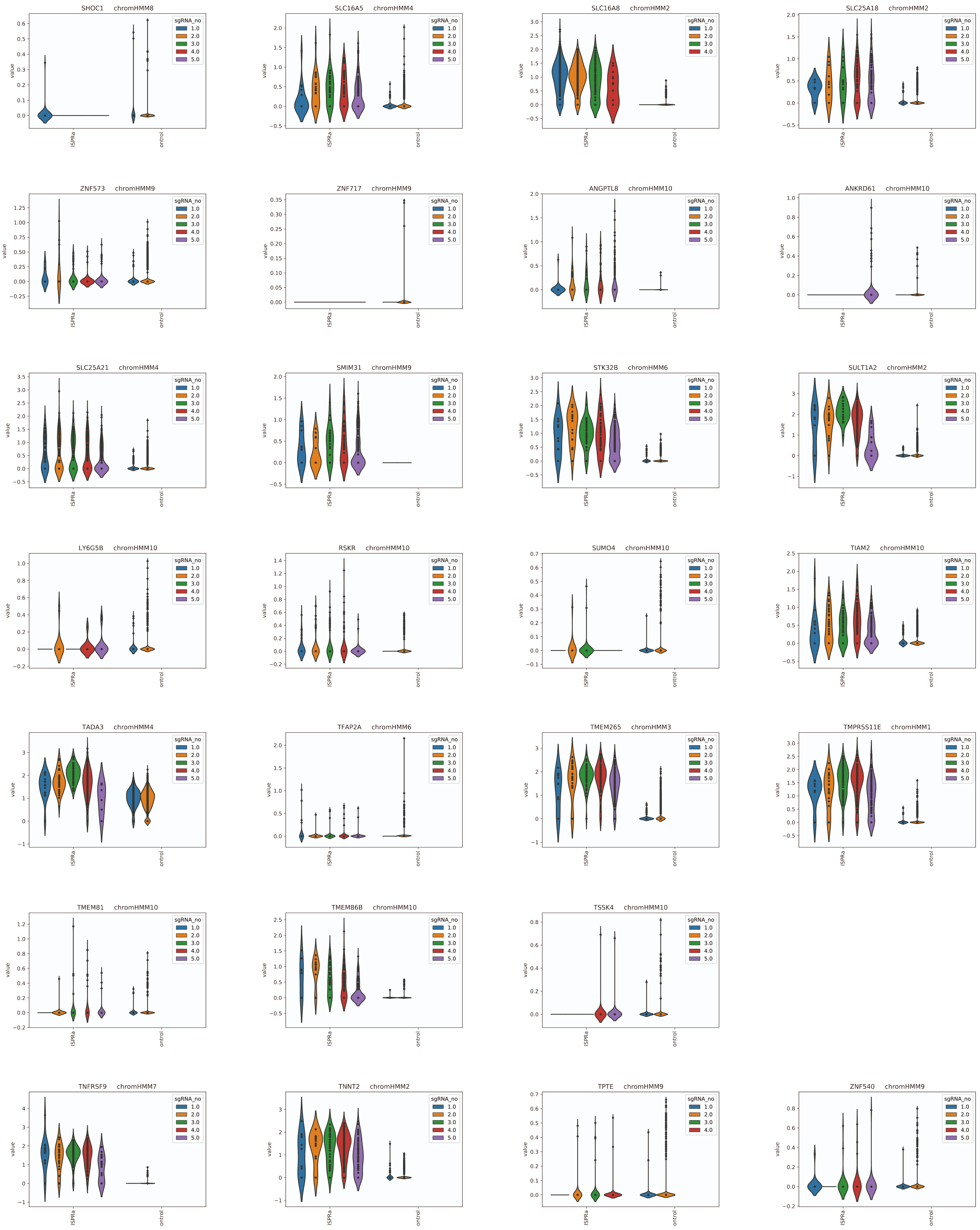
SHOC1 chromHMM8
SLC16A5 chromHMM4
SLC16A8 chromHMM2
SLC25A18 chromHMM2
ZNF573 chromHMM9
ZNF717 chromHMM9
ANGPTL8 chromHMM10
ANKRD61 chromHMM10
SLC25A21 chromHMM4
SMIM31 chromHMM9
STK32B chromHMM6
SULT1A2 chromHMM2
LY6G5B chromHMM10
RSKR chromHMM10
SUMO4 chromHMM10
TIAM2 chromHMM10
TADA3 chromHMM4
TFAP2A chromHMM6
TMEM265 chromHMM3
TMPRSS11E chromHMM1
TMEM81 chromHMM10
TMEM86B chromHMM10
TSSK4 chromHMM10
TNFRSF9 chromHMM7
TNNT2 chromHMM2
TPTE chromHMM9
ZNF540 chromHMM9
sgRNA_no
1.0
2.0
3.0
4.0
5.0
value
ISPRa
ontrol

Data S4

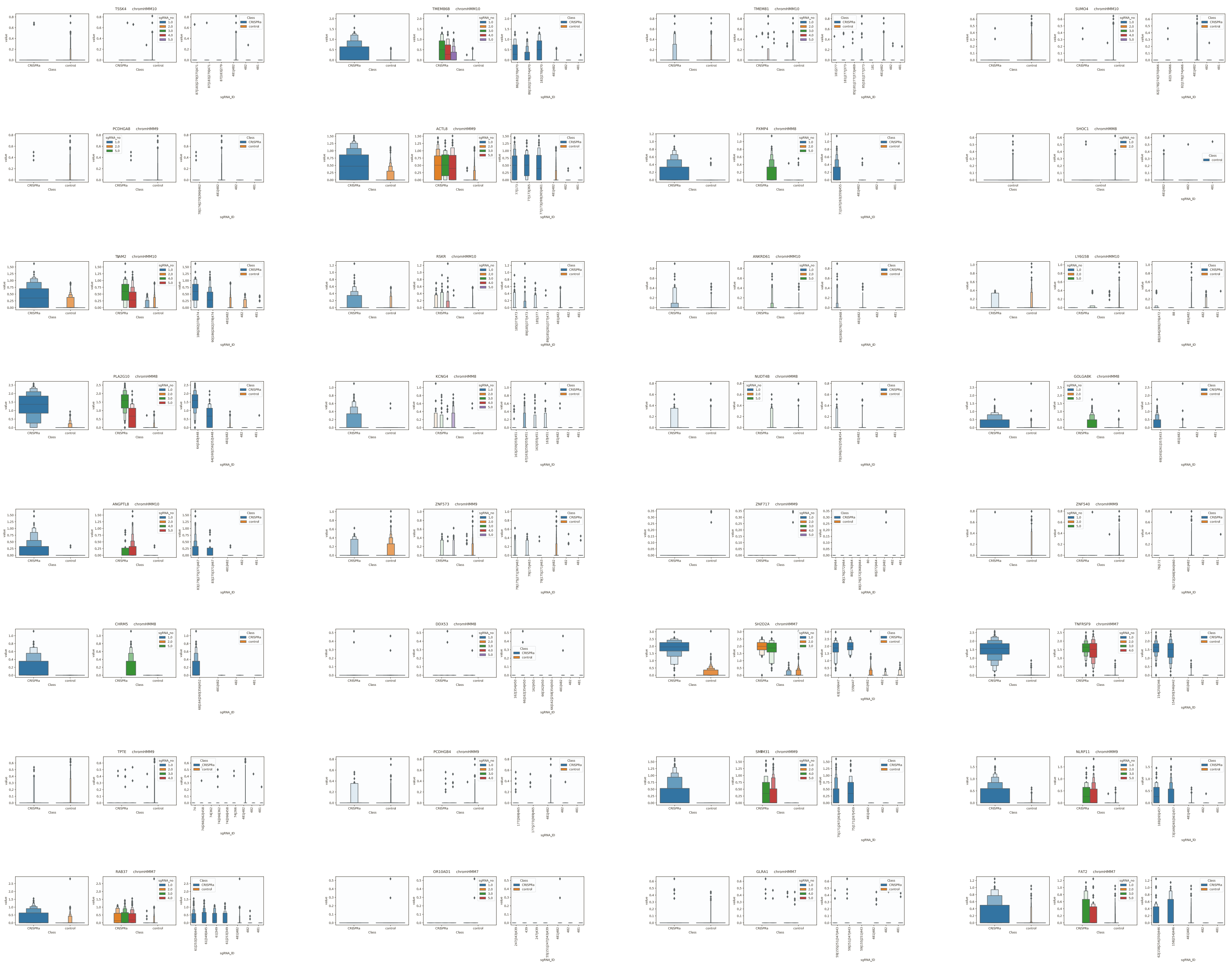

TSSK4 chromHMM10
TMEM86B chromHMM10
TMEM81 chromHMM10
SUMO4 chromHMM10
PCDHGA8 chromHMM9
ACTL8 chromHMM8
PXMP4 chromHMM8
SHOC1 chromHMM8
TIAM2 chromHMM10
RSKR chromHMM10
ANKRD61 chromHMM10
LY6G5B chromHMM10
PLA2G10 chromHMM8
KCNG4 chromHMM8
NUDT4B chromHMM8
GOLGA8K chromHMM8
ANGPTL8 chromHMM10
ZNF573 chromHMM9
ZNF717 chromHMM9
ZNF540 chromHMM9
CHRM5 chromHMM8
DDX53 chromHMM8
SH2D2A chromHMM7
TNFRSF9 chromHMM7
TPTE chromHMM9
PCDHGB4 chromHMM9
SMIM31 chromHMM9
NLRP11 chromHMM9
RAB37 chromHMM7
OR10AD1 chromHMM7
GLRA1 chromHMM7
FAT2 chromHMM7

Data S4

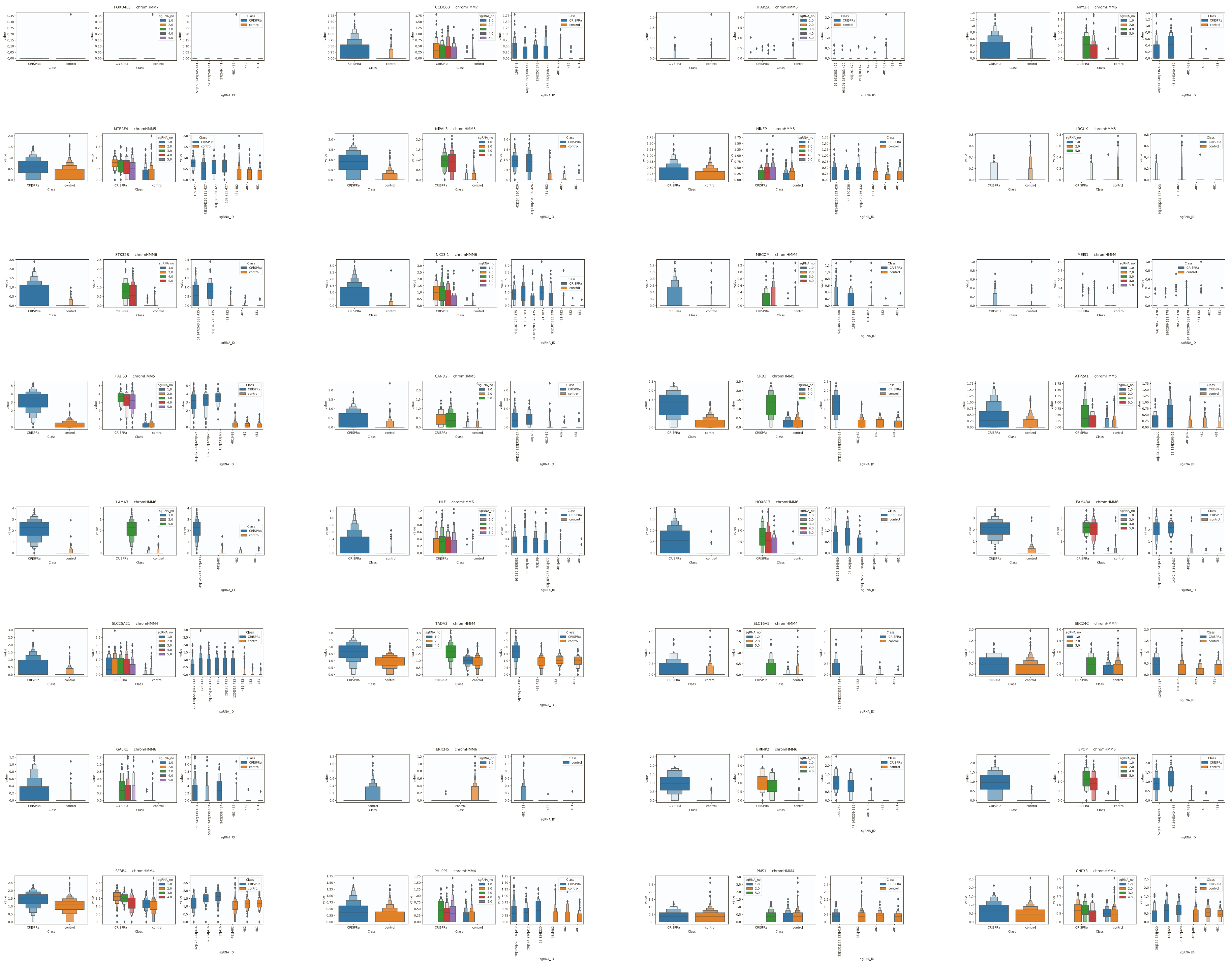

Data S4

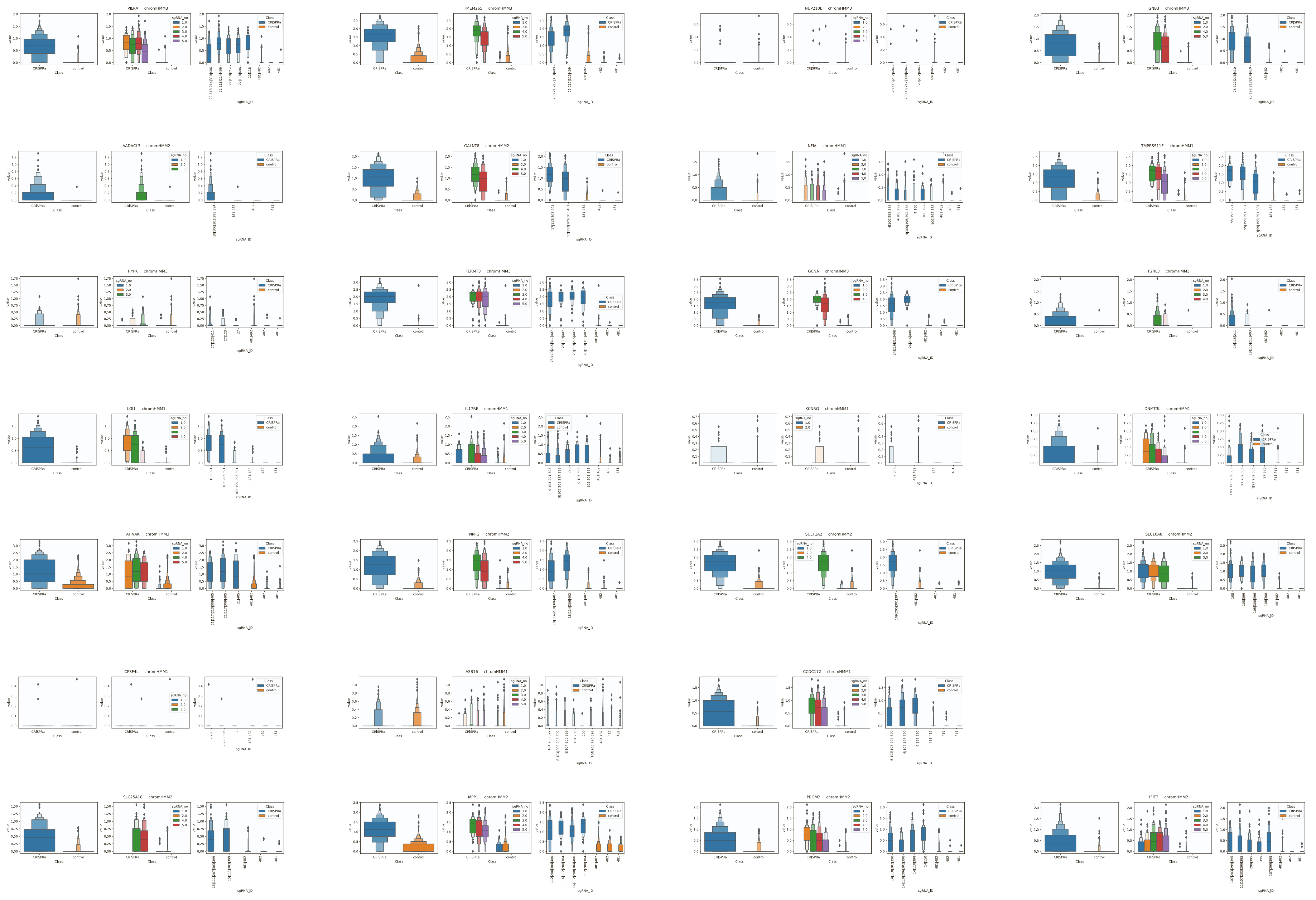

Supplement: Document S1. Figures S1–S5 and Data S1–S4 [file mmc1.pdf]
